# Targeting Fibroblast Activation Protein with [^177^Lu]Lu-FAP-2286 in Patients with Advanced Solid Tumors in the Phase I LuMIERE Trial

**DOI:** 10.1158/1078-0432.CCR-25-4356

**Published:** 2026-06-01

**Authors:** Jonathan McConathy, Vadim S. Koshkin, Yusuf Menda, Jordi Rodon, Ajit H. Goenka, Ryan H. Moy, Sophie Morse, Arnaud Demange, Lars Blumenstein, Burcu Babaoglan, Paola Aimone, Thomas A. Hope

**Affiliations:** 1 Department of Radiology, The University of Alabama at Birmingham, Birmingham, Alabama.; 2 Division of Hematology/Oncology, Department of Medicine, University of California San Francisco, San Francisco, California.; 3 Department of Radiology, University of Iowa, Roy J. and Lucille A. Carver College of Medicine, Iowa City, Iowa.; 4 Department of Investigational Cancer Therapeutics, The University of Texas MD Anderson Cancer Center, Houston, Texas.; 5 Mayo Clinic in Rochester, Rochester, Minnesota.; 6 Division of Hematology/Oncology, Department of Medicine, Columbia University Irving Medical Center, New York, New York.; 7 Novartis Pharmaceuticals Corp., East Hanover, New Jersey.; 8 Advanced Accelerator Applications, a Novartis Company, Geneva, Switzerland.; 9 Novartis Biomedical Research, Basel, Switzerland.; 10 Novartis Pharmaceuticals UK Ltd, London, United Kingdom.; 11 Novartis Pharma AG, Basel, Switzerland.; 12 Department of Radiology and Biomedical Imaging, University of California San Francisco, San Francisco, California.

## Abstract

**Purpose:**

Fibroblast activation protein (FAP) is an attractive target for radiopharmaceutical therapy. Phase I of the LuMIERE study (ClinicalTrials.gov, NCT04939610) investigated the safety of [^177^Lu]Lu-FAP-2286 (^177^Lu-FAP-2286) in heavily pretreated patients with advanced solid tumors and identified the recommended phase II dosage (RP2D).

**Patients and Methods:**

LuMIERE is a prospective, open-label, nonrandomized, phase I/II, multicenter study. Phase I followed a Bayesian optimal interval design evaluating four escalating activity levels of ^177^Lu-FAP-2286 (3.70, 5.55, 7.40, and 9.25 GBq). Patients were selected by positive [^68^Ga]Ga-FAP-2286 (^68^Ga-FAP-2286, also known as [^68^Ga]Ga-HKG301) PET/CT imaging on all target lesions [maximum standardized uptake value (SUV_max_) ≥1.5× SUV_mean_ of mediastinal blood pool]. ^177^Lu-FAP-2286 was administered intravenously every 6 weeks for ≤6 cycles. The primary endpoint was dosage-limiting toxicities (DLT) and treatment-emergent adverse events (TEAE).

**Results:**

Of 35 patients imaged with ^68^Ga-FAP-2286, 27 received ^177^Lu-FAP-2286 (3.70 GBq, *n* = 3; 5.55 GBq, *n* = 6; 7.40 GBq, *n* = 7; 9.25 GBq, *n* = 11). Two DLTs were observed: one patient who received 5.55 GBq of ^177^Lu-FAP-2286 experienced grade 4 lymphopenia, and one patient who received 9.25 GBq experienced grade 3 hemoptysis; these were the only treatment-related grade ≥3 TEAEs. All-cause grade ≥3 TEAEs were reported for 11 patients (40.7%). The RP2D for ^177^Lu-FAP-2286 monotherapy was determined to be 9.25 GBq. Preliminary Response Evaluation Criteria in Solid Tumors efficacy findings demonstrated a partial response in 1 patient and stable disease in 10 patients (maintained for at least two assessments in 6 patients).

**Conclusions:**

^177^Lu-FAP-2286 was well tolerated in patients with advanced solid tumors. The safety and preliminary efficacy findings support its continued development in phase II.

**Translational Relevance**

High fibroblast activation protein (FAP) expression in solid tumors compared with normal tissue makes FAP an attractive target for radiopharmaceutical therapies. FAP-2286 is a FAP-targeted ligand suitable for imaging and therapy. LuMIERE is a phase I/II study assessing [^177^Lu]Lu-FAP-2286 (^177^Lu-FAP-2286) in heavily pretreated patients with advanced solid tumors. Phase I demonstrated the utility of [^68^Ga]Ga-FAP-2286 imaging for selecting patients as ^177^Lu-FAP-2286 treatment candidates (*N* = 27) and established the safety profile of ^177^Lu-FAP-2286 monotherapy at activities up to 9.25 GBq. Treatment was well tolerated, with two dosage-limiting toxicities and infrequent, manageable hematologic adverse events. Preliminary organ dosimetry for single-cycle (9.25 GBq activity) and multicycle (≤55.5 GBq cumulative activity) administration was acceptable. Preliminary efficacy assessments were encouraging. Phase I findings from LuMIERE—the first multicenter assessment of the safety and tolerability of ^177^Lu-FAP-2286—support the investigation of ^177^Lu-FAP-2286 as a monotherapy and in novel combination regimens for various FAP-expressing solid tumors in phase II.

## Introduction

Cancer-associated fibroblasts (CAF) play a key role in creating an immunosuppressive tumor microenvironment by altering the function of innate/adaptive immune cells (1, 2), driving adaptive therapy resistance (1, 3), and remodeling the extracellular matrix to restrict drug penetration (4, 5). In the tumor stroma, CAFs mediate many aspects of tumor development and progression (3, 6–8). Paracrine signaling by CAFs (particularly through the release of TGFβ) regulates the epithelial-to-mesenchymal transition process, leading to an increase in tumor cell proliferative potential, higher mobility, and extracellular matrix remodeling (3, 7, 9). Fibroblast activation protein (FAP), a type II transmembrane serine protease, is highly expressed on the surface of CAFs present in the tumor microenvironment of many different cancer types (9–11), including breast, colorectal, pancreatic, lung, and ovarian tumors (9). With expression in more than 90% of carcinomas, FAP is considered a universal marker of CAFs (9). FAP is also expressed on the surface of some cancer cells (11). The low expression of FAP in normal tissues (9, 11–14) makes it a promising diagnostic and therapeutic target in a wide range of cancer types (15).

Given the continuing challenges of treatment resistance (16), novel treatment approaches are needed. For example, radioligand therapy—which has revolutionized the treatment of prostate cancer and some neuroendocrine tumors (17, 18)—could potentially target FAP, thus offering a valuable treatment option for patients with a range of different tumor types. Although radiolabeled inhibitors of FAP have been shown to be more sensitive and specific than conventional 2-deoxy-2-[^18^F]fluoro-D-glucose (^18^F-FDG) imaging for some cancer types (19), FAP-targeted radioligand imaging and radioligand therapy have the potential to deliver radiation to FAP-expressing CAFs in the stroma independent of the pharmacologic inhibitory function of the ligand. FAP-2286 is a molecule containing a cyclic peptide FAP ligand that confers high affinity for human FAP (11) and can be radiolabeled with ^68^Ga for diagnostic imaging or ^177^Lu for therapy (11). Preclinical models demonstrated rapid uptake of both [^68^Ga]Ga-FAP-2286 (^68^Ga-FAP-2286; also known as [^68^Ga]Ga-HKG301) and [^177^Lu]Lu-FAP-2286 (^177^Lu-FAP-2286) in FAP-expressing tumors (11). The delivery of beta radiation through the targeting of FAP-expressing CAFs with ^177^Lu-FAP-2286 is expected to disrupt the tumor stroma, change the tumor microenvironment, and result in the direct depletion of tumor cells by cross-fire radiation and/or binding to FAP-expressing cancer cells (11, 20). Clinical data from a prospective study in 21 patients with a range of cancers showed uptake of ^68^Ga-FAP-2286 in tumor lesions of all patients, regardless of tumor origin (epithelium or mesothelium) and whether the tumor was recurrent or metastatic (15). The first-in-human study of ^177^Lu-FAP-2286 demonstrated tumor uptake and retention in patients with FAP-expressing advanced adenocarcinomas (21).

LuMIERE is a phase I/II, open-label study and the first multicenter study to evaluate ^177^Lu-FAP-2286 in heavily pretreated patients with advanced solid tumors expressing FAP, as identified by ^68^Ga-FAP-2286 imaging. Phase I was designed to evaluate safety and establish the recommended phase II dosage (RP2D) for ^177^Lu-FAP-2286 monotherapy. This article reports the phase I results and describes preliminary dosimetry and efficacy data for ^177^Lu-FAP-2286.

## Patients and Methods

### Study design

**Figure 1.**
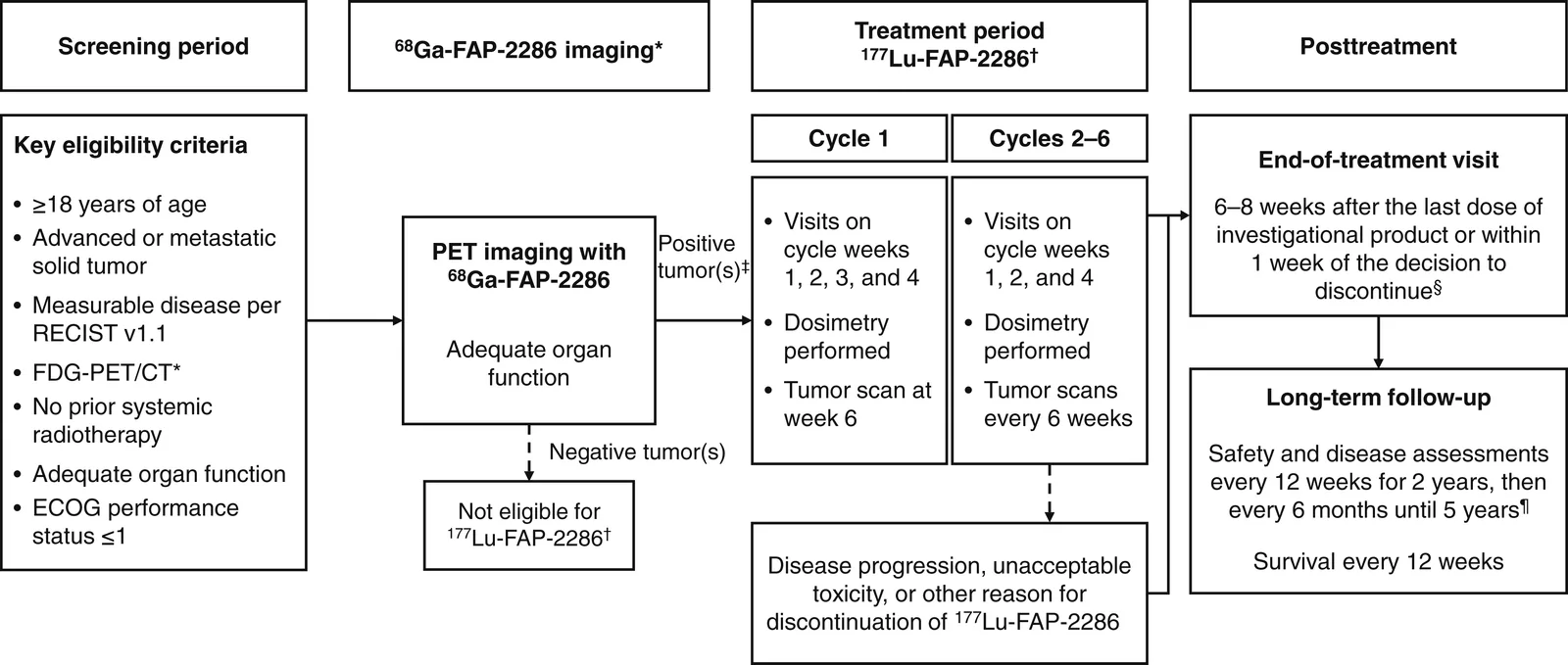
Theranostic workflow. *, FDG-PET/CT imaging was only performed for participants enrolled in phase I and could have been performed at any time during screening prior to cycle 1 day 1. This scan did not need to be repeated if the images were available from an FDG-PET/CT scan used for baseline disease assessments required for screening; ^†^, participants who did not have FAP-positive tumors and/or did not meet entry criteria for adequate organ function discontinued the study and did not proceed to the treatment phase of long-term follow-up but had safety assessments performed at the end-of-treatment visit; ^‡^, positive uptake was defined as all target lesions having ≥1.5× higher SUV_max_ compared with the SUV_mean_ of the mediastinal blood pool that was not attributable to other etiologies of tracer distribution; ^§^, the end-of-treatment visit occurred 6 to 8 weeks after the last dose of ^177^Lu-FAP-2286 or within 1 week after the decision to discontinue treatment for any reason other than participant withdrawal of consent from the study or death, including withdrawal of consent for treatment, withholding the last dose of ^177^Lu-FAP-2286, or not being eligible for continuation to ^177^Lu-FAP-2286 therapy following administration of ^68^Ga-FAP-2286; ^¶^, Safety assessments were performed until death or loss to follow-up. Disease assessments were performed until radiographic disease progression as assessed by the investigator, death, loss to follow-up, withdrawal from the study, study closure, or initiation of subsequent anticancer treatments. ECOG, Eastern Cooperative Oncology Group.

LuMIERE (ClinicalTrials.gov identifier NCT04939610, registered June 25, 2021) is an ongoing, prospective, phase I/II, multicenter, open-label, nonrandomized study. Phase I was conducted across six medical centers in the United States and utilized a Bayesian optimal interval (BOIN) design to guide activity escalation and determine the RP2D. The study was approved by an Institutional Review Board or independent ethics committee at each participating center and was performed in accordance with international guidelines (including the Declaration of Helsinki and the International Conference on Harmonisation Good Clinical Practice guidelines) and all applicable local regulations. All patients signed an approved informed consent form prior to participation. Protocol amendments pertinent to phase I are outlined in the Supplementary Methods. The theranostic workflow used in phase I of this study is shown in Fig. 1.

### Patient selection

Patients (aged ≥18 years) had (i) a histologically and/or cytologically confirmed advanced/metastatic solid tumor that was not amenable to treatment with curative intent and was either refractory to treatment or had progressed following prior treatment; (ii) measurable disease per Response Evaluation Criteria in Solid Tumors (RECIST) v1.1; (iii) adequate organ function (bone marrow, hepatic, and renal) prior to ^68^Ga-FAP-2286 administration; (iv) FAP expression on all target lesions, as determined by positive ^68^Ga-FAP-2286 uptake [maximum standardized uptake value (SUV_max_) ≥1.5× SUV_mean_ of the mediastinal blood pool] on PET/CT within 3 months prior to the first administration of ^177^Lu-FAP-2286; and (v) an Eastern Cooperative Oncology Group performance status of 0 or 1. Patients were excluded if they had (i) an active malignancy (except for the specific cancer under investigation in this study); (ii) symptomatic and/or untreated central nervous system (CNS) metastases, leptomeningeal disease, or a primary tumor of CNS origin (although patients with asymptomatic, previously treated CNS metastases were eligible provided they had been clinically stable for ≥4 weeks and had completed radiotherapy >2 weeks prior to treatment); (iii) received anticancer treatment with chemotherapy, antibody therapy (or other immunotherapy), angiogenesis inhibitors, vaccine therapy, gene therapy, or experimental drugs ≤14 days prior to the administration of ^177^Lu-FAP-2286 (or ≤28 days prior for checkpoint inhibitor therapy and other antibody therapies); or (iv) received radiopharmaceutical therapy or prior external beam radiation therapy (EBRT) to ≥25% of the bone marrow, prior EBRT directly to the kidney, or any EBRT within 2 weeks of the first scheduled ^177^Lu-FAP-2286 administration.

### PET/CT imaging

All patients had an FDG-PET/CT scan prior to cycle 1 day 1 using site standard processes and parameters. Patients were required to have a positive ^68^Ga-FAP-2286 PET/CT scan before ^177^Lu-FAP-2286 could be initiated. FAP-2286 was provided to investigational sites, and ^68^Ga-FAP-2286 was subsequently prepared by radiolabeling at the local radiopharmacy at each institution. Patients received an intravenous bolus administration of 111 to 296 MBq (±10%) of ^68^Ga-FAP-2286. For most patients [24/27 (89%)], PET imaging was performed approximately 60 minutes (±10 minutes) after injection; for some patients [3/27 (11%)], PET imaging was performed later (up to 120 minutes after injection). Per RECIST v1.1, target lesions (up to five per patient) required at least one linear dimension that could be accurately measured and was ≥10 mm in longest diameter in the axial plane (≥15 mm in the short axis for lymph node lesions).

Patients were considered eligible for treatment with ^177^Lu-FAP-2286 if the SUV_max_ of ^68^Ga-FAP-2286 in all target lesions was ≥1.5× the SUV_mean_ of the mediastinal blood pool. This was based on the quantitative analysis of ^68^Ga-FAP-2286 binding in tumors, for which the tumor SUV_max_ and SUV_mean_ were measured to calculate a tumor-to-background ratio (TBR; with blood pool SUV_mean_ used to define the background). Up to five tumor regions of interest (ROI) were generated to encompass the entire tumor uptake region of each of the site-selected target lesions. A threshold of 0.41× the SUV_max_ value within the tumor region was used as a starting point for segmentation. Manual edits were made to adjust for missed regions or removal of nuisance voxels, such as spillover from adjacent organs. The TBR was computed for each ROI. Specifically, for the blood pool SUV_mean_, a cylindrical ROI was generated within the lumen of the descending thoracic aorta, and the SUV_mean_ was computed as the mean SUV within the ROI. The tumor SUV_max_ computed from ^68^Ga-FAP-2286 PET/CT scans was compared with that computed from the FDG-PET/CT scans. Further details can be found in the Supplementary Methods.

### Treatment

^177^Lu-FAP-2286 was provided as a ready-to-use, single-activity vial and administered as a constant-rate intravenous infusion over approximately 30 minutes every cycle. At least 500 mL of saline was administered intravenously for approximately 2 hours following the infusion. ^177^Lu-FAP-2286 was administered every 6 weeks, up to a maximum of six administrations. The starting activity level was 3.70 GBq, with escalation in 1.85 GBq increments for the evaluation of four activity levels: 3.70, 5.55, 7.40, and 9.25 GBq. At least three, but no more than six, patients were evaluated at the starting activity level of 3.70 GBq. A dose cohort review committee decided whether to escalate the activity level, remain at the current activity level (maximum of 12 patients), or de-escalate the activity level based on the observed dosage-limiting toxicity (DLT) rate and the boundaries defined by the BOIN design. For the purpose of activity-escalation decisions, only DLTs occurring during the DLT period (≤42 days following the first administration of ^177^Lu-FAP-2286) were assessed. Proposed cumulative absorbed dose limits were 23 Gy in the kidneys or 2 Gy in the bone marrow, based on the thresholds established for EBRT and radioiodine therapy, respectively. Treatment was stopped in any patient at risk of crossing either threshold.

### Endpoints

The primary objective of phase I was to evaluate the safety and tolerability of ^177^Lu-FAP-2286 and determine the RP2D. The primary endpoints were DLTs, adverse events, serious adverse events, and clinical laboratory abnormalities. DLTs were defined as adverse events or abnormal laboratory values that were at least possibly related to treatment (as assessed by the investigator) and met prespecified toxicity/severity criteria (Supplementary Table S1). Secondary objectives (and corresponding endpoints) of phase I included evaluating the safety and tolerability of ^68^Ga-FAP-2286 (per adverse events and serious adverse events); determining tumor uptake using ^68^Ga-FAP-2286 (through SUV measures in tumor lesions); evaluating tumor uptake of ^68^Ga-FAP-2286 compared with ^18^F-FDG (by comparing SUV_max_ in target lesions); measuring the dosimetry of ^177^Lu-FAP-2286 (by estimating absorbed dose in organs); and evaluating the preliminary efficacy of ^177^Lu-FAP-2286 in advanced solid tumors (by investigator-assessed objective response per RECIST v1.1). This article does not report results relating to other phase I secondary objectives/endpoints (i.e., measurement of ^177^Lu-FAP-2286 dosimetry in tumor lesions and evaluation of its pharmacokinetic properties).

### Assessments

Safety was assessed continuously during treatment (at each scheduled visit after each administration of ^177^Lu-FAP-2286; on weeks 1 to 4 of cycle 1; on weeks 1, 2, and 4 of cycle 2; and on weeks 1 and 4 of subsequent cycles) and during follow-up. This schedule was selected to capture early postinfusion events and the expected timing of hematologic and organ toxicities observed with lutetium-labeled radioligand therapy. More frequent laboratory monitoring was performed during cycle 1 when first-cycle cytopenias and renal or urinary effects are most informative for assessing individual tolerability. Adverse events were classified using the Medical Dictionary for Regulatory Activities classification system (v25.1); severity was graded according to Common Terminology Criteria for Adverse Events (v5.0).

For the assessment of dosimetry, a hybrid imaging approach was initially mandated; whole-body planar scans were obtained at 4, 24, 48, and 168 hours, and single-photon emission computed tomography (SPECT)/CT scans were obtained at 24 and 168 hours following each infusion of ^177^Lu-FAP-2286. For later patient cohorts, the requirement for whole-body planar scans was removed, and dosimetry was assessed only by SPECT/CT scans on days 1, 2, 3, and 8 of each cycle (i.e., at 4, 24, 48, and 168 hours after infusion of ^177^Lu-FAP-2286). SPECT/CT parameters are described in the Supplementary Methods and Supplementary Table S2. SPECT/CT image data were preprocessed using VivoQuantTM software (VQ2020 or later). ROIs were drawn on each SPECT image by a trained image analyst. Calculation of the time-integrated activity coefficient (TIAC) was performed for each delineated organ and for up to five tumors for each patient. For each ROI and image acquisition (SPECT/CT), the concentration (Bq/mL), total activity (Bq), and ROI volume (mL) over time were computed with and without decay of ^177^Lu (a half-life of 9,662.4 minutes was used). End of infusion time was used for decay correction. Data were analyzed using multiexponential time–activity curve (TAC) fitting. Biological clearance of the tracer was derived from a data-appropriate fit of each organ TAC or via established biokinetic models for organs that did not exhibit exponential clearance. Depending on the shape and number of time points of the TAC, the data were fitted with the sum of one or two exponentials or using a rise-and-fall exponential function. The appropriate function was selected based on Akaike information criterion analysis and visual inspection by an imaging scientist or senior analyst. TIACs were calculated through the sum of trapezoidal integration for nonfitted parts of the TAC and analytic integration of the fitting curve from the start of the fit to infinity. The image-derived and gamma counter–derived TIACs were collated and input into Organ Level Internal Dose Assessment (OLINDA) 2.2.3 for the computation of organ and whole-body absorbed doses in addition to effective dose. For bone marrow dosimetry, a standard value for the red marrow-to-blood activity concentration ratio of 1.0 was used to estimate the TIAC of the bone marrow from whole blood concentration. Total red marrow mass was taken from the OLINDA ICRP-89 male or female model (as appropriate) and scaled to patient mass. Radiation exposure was closely monitored to ensure that the cumulative absorbed dose to the kidneys and bone marrow did not exceed 23 and 2 Gy, respectively. Further details can be found in the Supplementary Methods, including Supplementary Tables S3 and S4.

For the assessment of efficacy, tumor assessment scans (CT/MRI imaging of the chest, abdomen, and pelvis) were performed at screening/baseline (within 28 days of the start of study treatment) and prior to each administration of ^177^Lu-FAP-2286 during the treatment period (i.e., every 6 weeks starting from day 1 of cycle 1) until radiographic disease progression, death, loss to follow-up, or withdrawal of consent. Target and nontarget lesions were evaluated for evidence of radiographic response based on RECIST v1.1 criteria, as assessed by the investigator. Further details are included in the Supplementary Methods, including Supplementary Tables S5 and S6.

### Statistical analyses

Phase I of LuMIERE utilized a BOIN design to guide activity escalation and identify the single-cycle maximum tolerated activity (MTA) over the tested activity levels (i.e., the highest administered activity not expected to cause DLTs in more than 30% of patients during the DLT period). The target maximum sample size was 30 DLT-evaluable patients (or 12 DLT-evaluable patients at a given activity level); however, a minimum of three patients were treated prior to activity-escalation decisions. Assessment of DLTs was limited to the subset of patients who received the first scheduled administration of ^177^Lu-FAP-2286 in cycle 1 and either completed cycle 1 or experienced a DLT prior to completion of cycle 1. Safety analyses of ^68^Ga-FAP-2286 and ^177^Lu-FAP-2286 included all patients who received at least one administration of ^68^Ga-FAP-2286 and ^177^Lu-FAP-2286, respectively. Dosimetry analyses included all patients who received at least one administration of ^177^Lu-FAP-2286 and had sufficient serial image data for dosimetry determination. Efficacy analyses included all patients with measurable disease who received at least one administration of ^177^Lu-FAP-2286. Data were analyzed using SAS v9.4 (RRID:SCR_008567) and summarized using descriptive statistics; no formal statistical comparisons were performed.

## Results

### Patient disposition and characteristics

**Figure 2.**
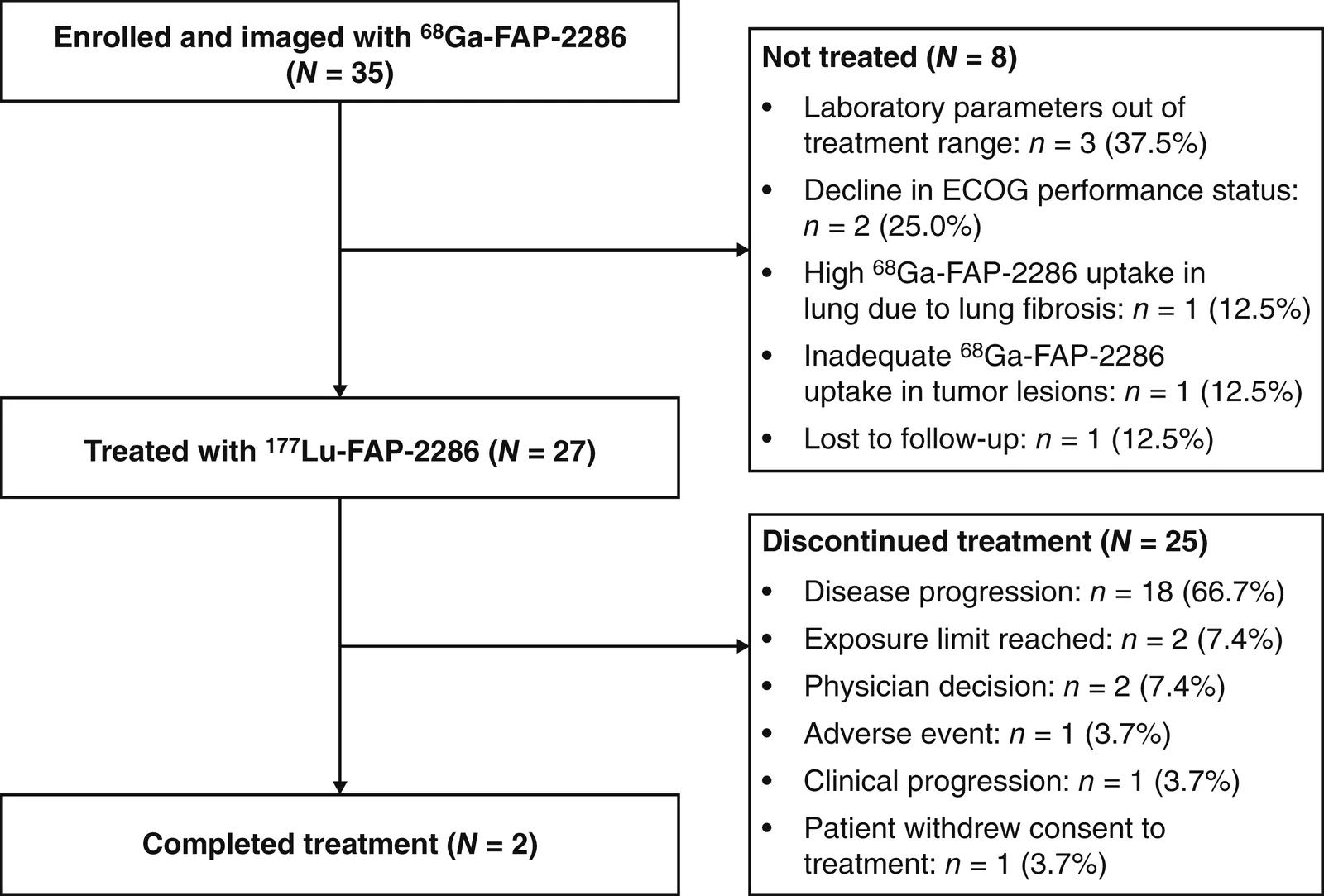
Patient disposition.

In phase I, 35 patients were enrolled between July 30, 2021, and October 19, 2023, and imaged with ^68^Ga-FAP-2286; 27 patients were treated with ^177^Lu-FAP-2286 across four activity levels (Fig. 2). Among the 27 patients treated, the median age was 60 years, and 51.9% were male (Table 1; Supplementary Table S7). The most common indications were pancreatic cancer (*n* = 9; 33.3%), soft-tissue sarcoma (*n* = 5; 18.5%), and colorectal cancer (*n* = 5; 18.5%; Table 1). All patients had received prior therapy; the mean number of previous therapies was 4.3 (Table 1). At the time of the data cutoff (March 24, 2025), two patients had completed all six cycles of treatment, and 25 had discontinued treatment.

**Table 1.** Baseline demographic and disease characteristics (^177^Lu-FAP-2286 safety set, *N* = 27).

Baseline characteristic	All patients
Age (years), median (range)	60 (25–75)
Sex, *n* (%)	​
Male	14 (51.9)
Female	13 (48.1)
Race^a^, *n* (%)	​
White	17 (63)
Black or African American	6 (22.2)
Asian	3 (11.1)
Not reported	1 (3.7)
ECOG performance status score, *n* (%)	​
0	10 (37)
1	16 (59.3)
Not reported	1 (3.7)
Indication, *n* (%)	​
Appendiceal cancer	1 (3.7)
Colorectal cancer	5 (18.5)
Gallbladder cancer	1 (3.7)
Head and neck cancer	3 (11.1)
Peritoneal mesothelioma cancer	1 (3.7)
Pancreatic cancer	9 (33.3)
Prostate cancer	1 (3.7)
Solitary fibrous cancer	1 (3.7)
Soft-tissue sarcoma^b^	5 (18.5)
Number of prior lines of therapy, mean (SD)	4.3 (1.4)

a Race was self-reported by patients; data were collected within electronic case report forms.

b Three cases of leiomyosarcoma and two cases of desmoplastic small round cell tumor.

### ^68^Ga-FAP-2286 safety

Of the 35 patients who underwent imaging with ^68^Ga-FAP-2286, one patient (2.9%) had a treatment-emergent adverse event (TEAE) within 2 days of receiving ^68^Ga-FAP-2286; this patient had a grade 3 small intestine obstruction that was not considered related to the study treatment.

### ^68^Ga-FAP-2286 and ^18^F-FDG uptake

In terms of ^68^Ga-FAP-2286 uptake, the median SUV_max_ of the 89 target lesions according to central assessment was 7.6 (range, 1.9–16.7) in the 27 patients treated with ^177^Lu-FAP-2286; the mean [standard deviation (SD)] SUV_max_ was 8.2 (3.8); and the mean (SD) TBR was 6.1 (2.8).

Similar uptake was observed with ^18^F-FDG. The median SUV_max_ of the 89 target lesions according to central assessment was 7.6 (range, 1.6–37.4) in the 27 patients treated with ^177^Lu-FAP-2286; the mean (SD) SUV_max_ was 9.6 (6.7); and the mean (SD) TBR was 5.7 (5.2). An exploratory analysis of ^18^F-FDG versus ^68^Ga-FAP-2286 uptake did not show a meaningful correlation (data not shown).

Nontumor uptake of ^68^Ga-FAP-2286 was observed in six patients. Diffuse uptake was observed in the lung of a patient who was subsequently diagnosed with pulmonary fibrosis and in the pancreas of another patient; both patients were excluded from treatment with ^177^Lu-FAP-2286 (though the latter was excluded on the basis of laboratory measurements being out of the acceptable range for patient eligibility). The remaining four patients received treatment with ^177^Lu-FAP-2286. This included two patients (one of whom had uterine fibroids) with diffuse uptake in the uterus, one with diffuse uptake in gastrointestinal tissue, and one with diffuse uptake in the left shoulder.

One patient with neuroendocrine medullary carcinoma was not eligible for ^177^Lu-FAP-2286 treatment due to inadequate ^68^Ga-FAP-2286 uptake in tumor lesions.

### ^177^Lu-FAP-2286 exposure

Up to six cycles of ^177^Lu-FAP-2286 were permitted. Of the 27 patients treated, 19 received only one cycle of ^177^Lu-FAP-2286. The majority of patients at each activity level also received a single cycle: two of three patients at activity level 1 (3.70 GBq), five of six patients at activity level 2 (5.55 GBq), four of seven patients at activity level 3 (7.40 GBq), and eight of eleven patients at activity level 4 (9.25 GBq). Overall, 5 of the 27 patients treated received two to four cycles, and 3 received five or six cycles (Table 2).

**Table 2.** ^177^Lu-FAP-2286 exposure.

Number of ^177^Lu-FAP-2286 administrations	3.7 GBq (*n* = 3) *n* (%)	5.55 GBq (*n* = 6) *n* (%)	7.4 GBq (*n* = 7) *n* (%)	9.25 GBq (*n* = 11) *n* (%)	Overall (*N* = 27) *n* (%)
1	2 (66.7)	5 (83.3)	4 (57.1)	8 (72.7)	19 (70.4)
2	0	0	0	2 (18.2)	2 (7.4)
3	0	0	2 (28.6)	0	2 (7.4)
4	0	1 (16.7)	0	0	1 (3.7)
5	0	0	1 (14.3)	0	1 (3.7)
6	1 (33.3)	0	0	1 (9.1)	2 (7.4)

### DLTs and RP2D

The estimated maximum cumulative activity levels (based on conservative EBRT thresholds) resulted in no further activity escalation, and the MTA for a six-cycle regimen was not reached in this study.

In total, two DLTs were observed. The first case was a grade 4 lymphopenia following the administration of ^177^Lu-FAP-2286 at activity level 2 (5.55 GBq) in cycle 1 (day 22). This patient, a 68-year-old male with stage IV head and neck cancer at study entry, had received five prior lines of therapy plus palliative radiation and presented with grade 2 lymphopenia at screening. There were no clinically relevant consequences of this DLT. The second case was a grade 3 hemoptysis following the administration of ^177^Lu-FAP-2286 at activity level 4 (9.25 GBq) in cycle 1 (day 14). This patient, a 72-year-old female with stage IV uterine leiomyosarcoma, had received seven prior lines of therapy plus palliative radiation and was receiving anticoagulants for atrial fibrillation at screening. A CT scan revealed lung metastases containing pseudoaneurysms (which were retrospectively identified on pretreatment imaging), and the patient’s anticoagulation medication was discontinued. The DLT was self-limiting, and no further intervention was required.

As the DLT rate for the 11 patients evaluated at activity level 4 (9.25 GBq) was below the prespecified acceptable toxicity threshold, and the activity level was already at the preplanned maximum for phase I, 9.25 GBq was selected as the RP2D.

### Safety

TEAEs (Table 3) were reported for all patients (*n* = 27; 100%); 14 (51.9%) had treatment-related TEAEs. Grade ≥3 TEAEs were reported for 11 patients (40.7%); of these, two (7.4%) had TEAEs that were considered treatment-related (lymphopenia, *n* = 1; hemoptysis, *n* = 1). Overall, seven patients (25.9%) experienced serious adverse events, with one (3.7%; hemoptysis) being determined to be treatment-related (Supplementary Table S8). In terms of clinical laboratory abnormalities (Supplementary Fig. S1), across all activity levels, there were no grade ≥3 decreases in hemoglobin, neutrophils, leukocytes, or platelets. Grade ≥3 decreases in lymphocytes were noted but were not associated with clinical symptoms or other clinically relevant findings.

**Table 3.** TEAEs occurring in ≥3 patients (all grades) or ≥1 patient (grade ≥3; ^177^Lu-FAP-2286 safety set, *N* = 27).

Preferred term	All grades, *n* (%)		Grade ≥3, *n* (%)	
	All causality	Treatment-related	All causality	Treatment-related
Patients with ≥1 TEAE	27 (100)	14 (51.9)	11 (40.7)	2 (7.4)
Fatigue	7 (25.9)	3 (11.1)	0	0
Anemia	6 (22.2)	4 (14.8)	0	0
Abdominal pain	5 (18.5)	0	3 (11.1)	0
Arthralgia	5 (18.5)	1 (3.7)	0	0
Diarrhea	5 (18.5)	2 (7.4)	0	0
Dyspnea	5 (18.5)	0	1 (3.7)	0
Ascites	4 (14.8)	0	2 (7.4)	0
Blood bilirubin increased	4 (14.8)	0	1 (3.7)	0
Weight decreased	4 (14.8)	0	0	0
Back pain	3 (11.1)	0	1 (3.7)	0
Constipation	3 (11.1)	0	0	0
Hyperkalemia	3 (11.1)	0	0	0
Hypoalbuminemia	3 (11.1)	0	0	0
Hypokalemia	3 (11.1)	0	0	0
Nausea	3 (11.1)	1 (3.7)	0	0
Abdominal distension	2 (7.4)	0	1 (3.7)	0
Asthenia	2 (7.4)	0	1 (3.7)	0
Cancer pain	2 (7.4)	0	1 (3.7)	0
Cholangitis	2 (7.4)	0	2 (7.4)	0
Hyponatremia	2 (7.4)	0	1 (3.7)	0
Lymphopenia	2 (7.4)	1 (3.7)	1 (3.7)	1 (3.7)
Device occlusion	1 (3.7)	0	1 (3.7)	0
Hemoptysis	1 (3.7)	1 (3.7)	1 (3.7)	1 (3.7)
Pulmonary embolism	1 (3.7)	0	1 (3.7)	0
Renal infarction	1 (3.7)	0	1 (3.7)	0
Spinal compression fracture	1 (3.7)	0	1 (3.7)	0
Spontaneous bacterial peritonitis	1 (3.7)	0	1 (3.7)	0

Data show patients for whom a TEAE occurred within 42 days of ^177^Lu-FAP-2286 administration. Preferred terms are sorted by descending frequency for the “All grades, All causality” column.

### Dosimetry

In total, 24 patients were evaluable for dosimetry. Across all activity levels (*n* = 24), the overall mean (SD) observed absorbed dose coefficient following the first administration of ^177^Lu-FAP-2286 in cycle 1 was 0.042 (0.011) Gy/GBq for the red marrow, 0.49 (0.088) Gy/GBq for the urinary bladder wall, and 0.35 (0.17) Gy/GBq for the kidneys. No other organs showed significant uptake and radiation risk (Supplementary Table S9); all other absorbed dose coefficients were <0.1 Gy/GBq. Based on the preliminary results, the mean (SD) cumulative absorbed dose of ^177^Lu-FAP-2286 across all activity levels (*n* = 24) for a cumulative administered activity of 55.5 GBq (six administrations of 9.25 GBq) was 2.3 (0.63) Gy for the red marrow, 27.0 (4.9) Gy for the urinary bladder wall, and 19.0 (9.7) Gy for the kidneys.

The estimated maximum cumulative absorbed activity limit of 23 Gy for the kidneys was exceeded in two patients at activity level 3 (7.40 GBq): the first patient had received three cycles of treatment, and the second had received five cycles. Treatment with ^177^Lu-FAP-2286 was discontinued in both patients.

### Efficacy

**Figure 3.**
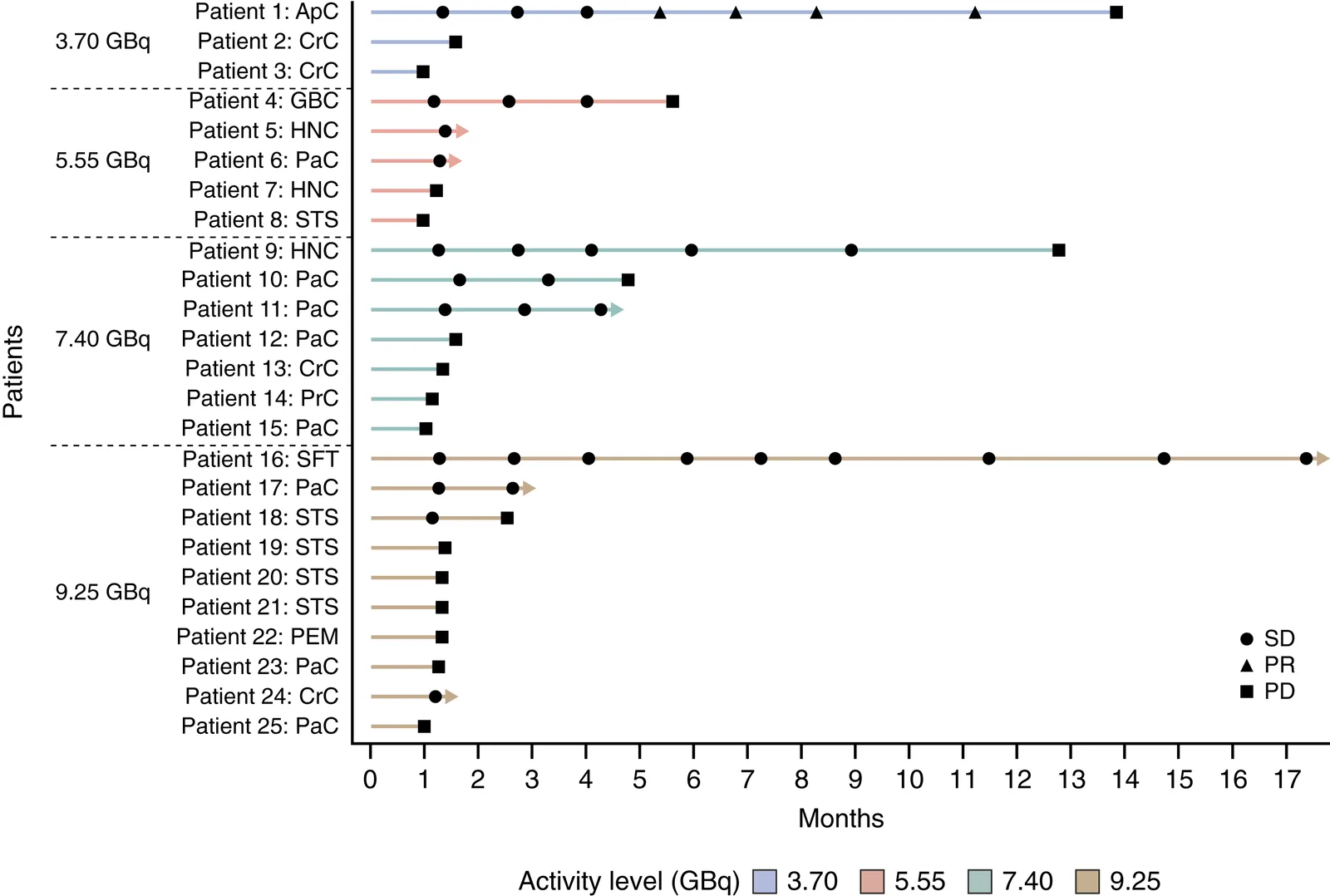
RECIST assessments over time (^177^Lu-FAP-2286 efficacy set, *N* = 27*). Arrows indicate patients who had not experienced disease progression at data cutoff (March 24, 2025). For STS, there were three cases of leiomyosarcoma and two cases of desmoplastic small round cell tumor. *Two patients discontinued prior to the first postbaseline RECIST assessment. ApC, appendiceal cancer; CrC, colorectal cancer; GBC, gallbladder cancer; HNC, head and neck cancer; PaC, pancreatic cancer; PD, progressive disease; PEM, peritoneal mesothelioma; PR, partial response; PrC, prostate cancer; SD, stable disease; SFT, solitary fibrous tumor; STS, soft-tissue sarcoma.

Overall, 25 patients had RECIST assessments. Best response included a partial response in one patient with appendiceal cancer who completed six cycles of ^177^Lu-FAP-2286 treatment (3.70 GBq). The partial response was observed after 5 months, with disease progression recorded at 14 months (Fig. 3). Ten patients achieved stable disease as the best response; these patients received different ^177^Lu-FAP-2286 activity levels (5.55 GBq, *n* = 3; 7.40 GBq, *n* = 3; 9.25 GBq, *n* = 4) and were diagnosed with a range of different cancer types (pancreatic cancer, *n* = 4; head and neck cancer, *n* = 2; colorectal cancer, *n* = 1; gallbladder cancer, *n* = 1; soft-tissue sarcoma, *n* = 1; solitary fibrous tumor, *n* = 1). Stable disease durations ranged from 1.5 months to >15 months (with some patients withdrawing consent or being lost to further follow-up). Ongoing stable disease (i.e., stable disease that was maintained over ≥2 RECIST assessments) was recorded in six patients at data cutoff, including one patient with solitary fibrous tumor who received six cycles of ^177^Lu-FAP-2286 (9.25 GBq) and had stable disease for >15 months. RECIST assessments by ^177^Lu-FAP-2286 activity level and tumor type are shown in Supplementary Fig. S2. A robust analysis of baseline ^68^Ga-FAP-2286 uptake and treatment response was not possible and will be explored in more detail in phase II.

## Discussion

In the phase I activity-escalation stage of the LuMIERE study, ^177^Lu-FAP-2286 was well tolerated in heavily pretreated patients with solid tumors, with a low incidence of DLTs. The RP2D for ^177^Lu-FAP-2286 monotherapy was determined to be 9.25 GBq (the highest preplanned activity level). Of the 27 patients assessed for preliminary efficacy, the best response was a partial response in one patient and stable disease in 10 patients.

During the imaging portion of the study, PET imaging with ^68^Ga-FAP-2286 successfully identified FAP-positive lesions in patients with various solid tumors, with ^68^Ga-FAP-2286 SUV_max_ ranging from 1.9 to 16.7. This result is consistent with the literature, which reports high uptake of ^68^Ga-FAP-2286 on PET/CT scans of patients with a wide variety of cancers (15, 22–24). Our results expand this published evidence and provide further support for ^68^Ga-FAP-2286 as a tool to identify FAP-positive solid tumors and select patients for FAP-targeted therapy. Although the current dataset shows only a weak correlation between ^18^F-FDG and ^68^Ga-FAP-2286 uptake, the patient population is small and heterogeneous, and future studies could explore this relationship in selected tumor types. The SUV_max_ threshold of ≥1.5× SUV_mean_ of the mediastinal blood pool was selected to maximize patient eligibility and facilitate the assessment of the safety profile of ^177^Lu-FAP-2286 across a heterogeneous group of patients. Further studies are needed to clarify the relationship between baseline ^68^Ga-FAP-2286 uptake and ^177^Lu-FAP-2286 tumor absorbed dose and subsequent efficacy and to establish optimal thresholds for patient selection.

For the treatment portion of the study, a dosing schedule of every 6 weeks was used. This regimen was considered reasonable based on other FDA-approved lutetium-labeled radioligand therapies [^177^Lu-vipivotide tetraxetan, every 6 weeks; [^177^Lu]Lu-DOTATATE (^177^Lu-DOTATATE), every 8 weeks; refs. 25, 26]. Overall, ^177^Lu-FAP-2286 treatment was well tolerated in heavily pretreated patients with solid tumors. Eleven patients (40.7%) experienced a grade ≥3 TEAE, with two (7.4%) considered related to treatment (also defined as DLTs). This aligns with the first-in-human study reported by Baum and colleagues (21), with administration of ^177^Lu-FAP-2286 at varying activities (mean 5.8 ± 2.0 GBq; range, 2.4–9.9 GBq), in which three grade ≥3 adverse events were reported among 11 patients (27.3%) with advanced adenocarcinomas in the rectum, pancreas, ovaries, and breast. In our study, two DLTs (grade 4 lymphopenia and grade 3 hemoptysis) were recorded among the 27 patients treated with ^177^Lu-FAP-2286. With respect to the grade 4 lymphopenia observed at 5.55 GBq, it should be noted that lymphopenia is common among patients who have been heavily pretreated with chemotherapy (27, 28), such as in the patient population studied, and that the patient in question presented with grade 2 lymphopenia at screening. In addition, hematologic toxicities, including lymphopenia, have been observed with radioligand therapy but are often transient and do not require clinical intervention (29–31). The grade 3 hemoptysis observed at 9.25 GBq may have been due to disease progression in known lung metastases containing retrospectively identified pseudoaneurysms in the context of the patient receiving anticoagulants. The low rate of cytopenias observed with ^177^Lu-FAP-2286 treatment, even in a heavily pretreated population, is encouraging and opens the possibility of combination therapies, particularly with chemotherapy. Disruption of the tumor microenvironment by ^177^Lu-FAP-2286 (20) may improve cytotoxic chemotherapy delivery (32, 33), and, in turn, chemotherapy may sensitize tumors to the effects of radiation (34), thus offering a potential multimodal approach to tumor destruction. Consequently, treatment with ^177^Lu-FAP-2286 in combination with chemotherapy will be assessed further in the phase II part of LuMIERE. Future studies could explore other potential ^177^Lu-FAP-2286 combination approaches, aiming to improve efficacy with nonoverlapping safety profiles (20, 34–39). Overall, the manageable safety of ^177^Lu-FAP-2286 in phase I, including the low rate of cytopenias, supports the administration of the RP2D every 4 weeks for up to six cycles in phase II. The increased frequency of this administration schedule (relative to phase I) may lead to increased response rates and reduce the opportunity for tumor regrowth between treatment cycles.

For all activities of ^177^Lu-FAP-2286 investigated (3.70–9.25 GBq), the absorbed doses in organs did not raise any clinical concerns and were within accepted tolerance limits (although two patients discontinued treatment to avoid exceeding the exposure limits). The mean absorbed dose coefficients for the kidneys (0.35 Gy/GBq) and red marrow (0.042 Gy/GBq) following the first administration of ^177^Lu-FAP-2286 in this study were in line with or lower than those previously reported by Baum and colleagues (21), who reported mean absorbed doses of 1.0 Gy/GBq for the kidneys and 0.05 Gy/GBq for the red marrow. Although cross-study comparison is difficult, the kidney dose coefficient of ^177^Lu-FAP-2286 (0.35 Gy/GBq) compares favorably with that observed with ^177^Lu-DOTATATE in NETTER-1 (0.65 Gy/GBq) and [^177^Lu]Lu-PSMA-617 in VISION (0.43 Gy/GBq; refs. 40, 41). Dosimetry data for the urinary bladder wall were consistent with preclinical data indicating that ^177^Lu-FAP-2286 elimination is primarily via renal excretion (11). In this study, postinfusion intravenous hydration was mandated. Future strategies, such as more frequent voiding protocols and hydration regimens, could be explored to reduce the relatively high cumulative absorbed dose observed in the urinary bladder wall (27.0 Gy) if indicated. However, it should be noted that no significant urinary treatment-related adverse events were observed at the RP2D. For a cumulative activity of 55.5 GBq, the mean (SD) cumulative absorbed dose by the kidneys was 19.0 Gy (9.7) and below the EBRT-defined threshold of 23 Gy (42). This renal threshold prevented further activity escalation for a six-cycle regimen although the MTA was not reached, suggesting this is conservative for radioligand therapy and is not considered definitive. The low red marrow dose coefficient (0.042 Gy/GBq) is encouraging. The mean (SD) cumulative dose to the bone marrow was 2.3 Gy (0.63), slightly higher than the 2 Gy threshold (43), but minimal hematologic toxicity was observed. The bone marrow threshold has been extrapolated from studies with ^131^Iodine therapy and represents an acute limit following one administration rather than a cumulative long-term threshold. Its relevance to radioligand therapy with lutetium has been questioned (44–46). Additionally, Strosberg and colleagues (47) argue against extrapolating EBRT constraints to radioligand therapy and recommend using complete blood count as an alternative means of assessing bone marrow toxicity. As noted by Strosberg and colleagues (47), bone marrow dosimetry lacks precision and may vary depending on patient characteristics and the radionuclide used; furthermore, research shows that the absorbed dose in bone marrow does not correlate strongly with cytopenias observed in patients (44, 48, 49). Overall, the risk/benefit profile of ^177^Lu-FAP-2286 allows for activity escalation to be considered.

Although most patients who received ^177^Lu-FAP-2286 in phase I of the LuMIERE study experienced disease progression, this is not unexpected for such a heavily pretreated patient population with limited treatment options. In addition, the administration frequency of ^177^Lu-FAP-2286 may have allowed tumor regrowth, and the low ^68^Ga-FAP-2286 uptake eligibility criterion may have led to low tumor absorbed doses. Nevertheless, preliminary efficacy signals were observed across a range of tumor types. Partial response was reported in one patient with appendiceal cancer, and stable disease was reported in 10 patients, among whom the most durable response was observed for a patient with solitary fibrous tumor. The results described here are consistent with several case studies that reported responses to ^177^Lu-FAP-2286 in metastatic tumors, including lung, bladder, and breast cancers, as well as in rarer cancers such as bone malignant solitary fibrous tumor, ovarian neuroendocrine tumors, and rhabdoid meningioma (50–57). The results from these single cases are encouraging, but robust confirmatory clinical studies, such as the LuMIERE phase II trial, are needed to determine the efficacy of this agent. Phase II of LuMIERE will further investigate the efficacy of ^177^Lu-FAP-2286 and will enroll approximately 138 patients (identified by ^68^Ga-FAP-2286 imaging) to evaluate ^177^Lu-FAP-2286 monotherapy in pancreatic ductal adenocarcinoma, non–small cell lung cancer, and breast cancer (as separate groups), as well as ^177^Lu-FAP-2286 in combination with chemotherapy in pancreatic ductal adenocarcinoma and non–small cell lung cancer (also as separate groups). These tumor types were selected because the tumor microenvironments have elevated FAP expression (58, 59), which has been correlated with poor survival—especially in later-line settings, in which there is an urgent need for new treatment options.

Our study has some limitations. Inherent to the design of phase I studies, the number of patients in each activity cohort was small and included diverse tumor types, making it difficult to draw definitive conclusions about the efficacy or tumor-specific activity of ^177^Lu-FAP-2286. Of note, ^177^Lu-FAP-2286 is expected to both modulate the tumor microenvironment (via the targeting of FAP-expressing CAFs in the stroma) and directly deplete tumor cells (via direct and/or cross-fire effect; refs. 11, 20); however, the relative contribution of these mechanisms to the overall therapeutic effect is unclear. With this in mind, it is possible that the response to ^177^Lu-FAP-2286 could differ by tumor type due to variations in the relative distribution of FAP-expressing CAFs versus FAP-expressing tumor cells or due to variations in tumor vascularization and stromal density (affecting therapeutic access; refs. 60, 61). Another limitation is that efficacy assessments were based on investigator assessments, without central independent review, and the follow-up time was limited, especially for patients who were enrolled at the higher activity levels. Most patients had documented disease progression before the second administration of ^177^Lu-FAP-2286. As mentioned previously, this might be explained by the frequency of ^177^Lu-FAP-2286 administration allowing tumor regrowth and the low ^68^Ga-FAP-2286 uptake level used to determine eligibility for phase I, which is likely to have resulted in low tumor-absorbed doses. To maximize the likelihood of efficacy in phase II, ^177^Lu-FAP-2286 will be administered more frequently (every 4 weeks instead of every 6 weeks), and a more stringent ^68^Ga-FAP-2286 uptake threshold will be used to determine patient eligibility. This will allow exploration of any potential relationship between ^68^Ga-FAP-2286 uptake and the efficacy of ^177^Lu-FAP-2286.

Future research directions include characterizing the relationship between FAP expression on CAFs and tumor cells, tumor absorbed dose, and response to ^177^Lu-FAP-2286 therapy. In addition, the optimal threshold of ^68^Ga-FAP-2286 uptake for the selection of patients for treatment with ^177^Lu-FAP-2286 remains to be elucidated. Also, future studies could assess whether higher activity per cycle or more frequent administration improves the therapeutic index of ^177^Lu-FAP-2286. As already discussed, the cumulative dose thresholds frequently used in radioligand therapy studies are suboptimal, and further research is needed to understand the relationship between organ dosimetry and the observed tolerability profile. As the field develops and matures, it will be critical to understand the potential mechanisms of resistance to FAP-targeted radioligand therapies. This will inform both the rational development of further agents and the sequencing of treatment in clinical practice.

Overall, the safety findings from phase I of the LuMIERE study support the continued development of ^177^Lu-FAP-2286 in the phase II part of the study, which is investigating ^177^Lu-FAP-2286 as monotherapy and in combination with chemotherapy in pancreatic ductal adenocarcinoma, non–small cell lung cancer, and breast cancer.

## Supplementary Material

Supplementary Methods S1 Supplementary Methods.

Supplementary Figure S1 Figure S1. Clinical laboratory measurements over time by activity level.

Supplementary Figure S2 Figure S2. RECIST assessments over time (change from baseline in target lesions sum of diameters) by activity level and cancer type.

Supplementary Table S1 Table S1. Prespecified toxicity/severity criteria for dosage-limiting toxicities.

Supplementary Table S2 Table S2. SPECT/CT parameters.

Supplementary Table S3 Table S3. OLINDA source organs.

Supplementary Table S4 Table S4. OLINDA target organs.

Supplementary Table S5 Table S5. CT acquisition parameters.

Supplementary Table S6 Table S6. MRI acquisition parameters.

Supplementary Table S7 Table S7. Representativeness of study participants.

Supplementary Table S8 Table S8. Serious adverse events.

Supplementary Table S9 Table S9. Dosimetry of 177Lu-FAP-2286 in Cycle 1.

## Data Availability

Novartis is committed to sharing access to patient-level data and supporting clinical documents from eligible studies with qualified external researchers. These requests are reviewed and approved by an independent review panel on the basis of scientific merit. All data provided are anonymized to respect the privacy of patients who have participated in the trial, in line with applicable laws and regulations. This trial data availability is according to the criteria and process described on www.clinicalstudydatarequest.com.
