# Supplementary material for: Targeting Fibroblast Activation Protein with [177Lu]Lu-FAP-2286 in Patients with Advanced Solid Tumors in the Phase I LuMIERE Trial
Source: Clin Cancer Res. 2026 Jun 1;32(17):3757–67. doi: 10.1158/1078-0432.CCR-25-4356 (PMC13335097; doi:10.1158/1078-0432.CCR-25-4356)
Supplement: Supplementary file 1 [file ccr-25-4356_supplementary_figure_s1_suppfs1.docx]

**Supplementary data – Figure S1.** Clinical laboratory measurements over time by activity level (^177^Lu-FAP-2286 safety set, N=27): lymphocytes (**A**), neutrophils (**B**), leukocytes (**C**), hemoglobin (**D**), platelets (**E**), serum creatinine (**F**).

Markers indicate ^177^Lu-FAP-2286 infusion days for each patient.
