# Supplementary material for: Targeting Fibroblast Activation Protein with [177Lu]Lu-FAP-2286 in Patients with Advanced Solid Tumors in the Phase I LuMIERE Trial
Source: Clin Cancer Res. 2026 Jun 1;32(17):3757–67. doi: 10.1158/1078-0432.CCR-25-4356 (PMC13335097; doi:10.1158/1078-0432.CCR-25-4356)
Supplement: Supplementary file 2 [file ccr-25-4356_supplementary_figure_s2_suppfs2.docx]

**Supplementary data – Figure S2.** RECIST assessments over time (change from baseline in target lesions sum of diameters) by activity level and cancer type (^177^Lu-FAP-2286 efficacy set, N=25): 3.70 GBq (**A**), 5.55 GBq (**B**), 7.40 GBq (**C**), and 9.25 GBq (**D**).

PD indicates timepoints where an overall response of PD is reported, based on target, non-target, or new lesions.

Patients with no label had not experienced disease progression at data cutoff (March 24, 2025).

PD, progressive disease; RECIST, Response Evaluation Criteria in Solid Tumors.
