# Supplementary material for: Targeting Fibroblast Activation Protein with [177Lu]Lu-FAP-2286 in Patients with Advanced Solid Tumors in the Phase I LuMIERE Trial
Source: Clin Cancer Res. 2026 Jun 1;32(17):3757–67. doi: 10.1158/1078-0432.CCR-25-4356 (PMC13335097; doi:10.1158/1078-0432.CCR-25-4356)
Supplement: Supplementary file 3 [file ccr-25-4356_supplementary_methods_s1_suppms1.docx]

# Supplementary methods

## Protocol amendments

The following change was made to the study design:

- From: *Patients meeting entry criteria will be enrolled and will undergo PET imaging with ^68^Ga-FAP-2286 prior to initiating treatment with ^177^Lu-FAP-2286.*
- To: *Patients meeting entry criteria will be enrolled and patients who have not had prior PET/CT imaging with ^68^Ga-FAP-2286 in the previous 3 months will undergo PET imaging with ^68^Ga-FAP-2286 prior to initiating treatment with ^177^Lu-FAP-2286.*

This was to allow patients with qualifying prior PET imaging with ^68^Ga-FAP-2286 to screen and enroll without repeating this step.

The following change was made for imaging dosimetry:

- From: *All Phase 1 dose escalation patients will have planar scans at 4, 24, 48 and 168 hours and single-photon emission computerized tomography (SPECT)/CT scans at 24 and 168 hours after each dose in each cycle*.
- To: *All patients will have SPECT/CT scans at four time points, including scans on Day 1, Day 2, Day 3, and Day 8 of each cycle. The windows for the post-infusion SPECT/CT time points are: 4 hours (± 2 hours); 24 hours (± 4 hours), 48 hours (± 4 hours); and 168 hours (± 8 hours). All SPECT/CT scans should encompass 2 or more contiguous bed positions, such that all target lesions and both kidneys are imaged at each time point.*

This was to provide more accurate and quantifiable imaging data for dosimetry calculations, and to simplify the imaging schedule at clinical trial sites.

## ^68^Ga-FAP-2286 imaging acquisition

Prior to patient screening, all positron emission tomography/computed tomography (PET/CT) scanners were qualified and approved. The following scanner conditions were required:

- A dose calibrator that had been checked to ensure precision and accuracy.
- A method of determining the actual administered activity.
- Accurate clock times that recorded the exact times of dose calibration, dose administration, scan initiation, and scan completion. Clock times were synchronized with the PET scanner.
- The predefined acquisition protocol for the trial was selected.

The following steps were followed when scanning:

1. A CT scan was performed for attenuation correction using site standard CT parameters, before/after the emission acquisition, per institutional guidelines.
2. The PET acquisition started at 50 to 120 minutes after radiotracer administration.
3. PET body coverage included skull vertex to mid-thigh, unless there was tumor in the legs or feet. Patients with tumors in the lower limbs were imaged from the vertex of the skull to feet.
4. Scanning was conducted for 3 minutes per bed position, performed caudal-cranial and according to institutional standard protocol.
5. The number of bed positions, overlap, and other related parameters were dependent on patient size and consistent with the recommendations of the PET/CT scanner manufacturer.
6. The reconstruction was performed using an approved protocol that utilized an iterative reconstruction method and parameters (iterations, subsets, matrix, post-filtering) that were established during the site setup process. Both attenuation corrected and non-attenuation corrected PET files were required to be submitted, unless noted otherwise.

Parameters for SPECT/CT imaging are outlined in **Table S2**.

## ^177^Lu-FAP-2286 imaging procedures

^177^Lu-FAP-2286 imaging was acquired using multi-headed SPECT/CT scanners. For each patient, all imaging was completed on the same scanner. Only the specific SPECT/CT camera qualified and approved at the start of the study was used. SPECT/CT anatomical coverage encompassed two or more contiguous bed positions, such that all target lesions and both kidneys were imaged at each time point. SPECT/CT parameters are outlined in **Table S2**. The same image reconstruction parameters were used for all scans for the same patient. The reconstruction was performed using an iterative reconstruction method that was established and approved during the site qualification process. The approved reconstruction parameters (iterations, subsets, matrix, post-filtering, etc.) were kept consistent for all imaging time points.

## ^177^Lu reference standard

For each patient, a reference standard was prepared prior to the first ^177^Lu-FAP-2286 SPECT/CT scan of each cycle, by injecting approximately 3.7 MBq (100 μCi) of ^177^Lu into a container containing 100 mL of water and/or saline, and imaged with the patient, when possible. The radioactivity in the syringe used in preparing the standard was measured in the imaging center’s dose calibrator immediately prior to injecting into the reference standard container, and a residual assay of the syringe was measured immediately following injection to obtain an accurate net activity administered. One 0.5 mL sample was taken from the reference standard as part of the initial preparation. This sample was placed in a vacutainer tube (the same tubes used for patient blood collection) and filled with saline or water to achieve a total sample volume of 1 mL. The 1 mL sample was measured (counts per minute) using the approved gamma counter at the time of patient’s sample counting (blood, etc.). For SPECT/CT scans, the reference standard was taped or secured to the patient just below the navel.

If the reference standard could not be included in the patient scan, a separate scan containing the reference standard was acquired immediately following each patient SPECT/CT scan as a separate scan using the same acquisition (only one field of view [FOV] required) and reconstruction parameters as used for the patient scan. The entire reference standard was completely included within the gamma camera FOV for all scans to aid in subsequent dosimetry calculations.

## ^177^Lu-FAP-2286 SPECT/CT dosimetry analysis

### Image analysis

Imaging data were analyzed using VivoQuant software (VQ2020 or later). The general quantification approach was based on the principles detailed in Medical Internal Radiation Dose (MIRD) Pamphlet No. 16, No. 23 (1), and No. 26 (2), as appropriate and with some modifications, due to limitations in image data acquisition.

### SPECT calibration factor

A reference standard of known ^177^Lu activity placed in the SPECT FOV was used to compute a patient-specific calibration factor. It was ensured that the SPECT acquisition was consistent with the one determined at site set-up (within ±10%). The site set-up calibration factor was used unless the patient-specific calibration factor varied by more than ±10% from the site set-up calibration factor. In this instance, the patient’s specific calibration factor was used.

Correction for partial volume effect for lesions was done using recovery coefficients. Recovery coefficients were computed using a National Electrical Manufacturers Association (NEMA) International Electrotechnical Commission (or adequate) phantom with hot spheres and warm background scanned during the site qualification process. Phantoms were prepared according to the Instrumentation Set-up Manual. Phantom spheres were segmented using a spherical volume of interest (VOI) of the same diameter as the NEMA phantom physical sphere, and a region of interest (ROI [large sphere]) was drawn in the background region of the phantom to derive a calibration factor as described above. The activity concentration was derived for each ROI by multiplying the number of counts by the calibration factor calculated using the phantom background region. The recovery coefficient was determined by taking the activity concentration obtained from the SPECT measurement in each sphere divided by the true activity concentration in the sphere. The recovery coefficient was plotted as a function of sphere diameter. Data were fitted with the following function:

**RC(V)=A×In(V)−B**,

where V was the sphere volume, and A and B were fitting constants (A, scaling parameter, and B, offset or baseline). The function was used to determine the recovery coefficient to apply based on lesion size.

### Organ ROI and lesion delineation

An Image Analysis specialist performed a visual assessment of the SPECT/CT images and assessed the organs included in the International Commission on Radiological Protection male or female (as appropriate) adult (ICRP)-89 derived phantom (**Table S3**). For all organs showing radionuclide uptake above background, an ROI was generated. Where possible, ROI delineation methods were kept consistent across the study patients and image types. Variations in ROI delineation were sometimes needed to avoid portions of organs that contained metastasis or anatomical variances between patients. Organ ROIs were reviewed by a senior image analyst (no radiologist review). Up to five tumor ROIs per patient were drawn for the tumors based on site-defined lesions. A nuclear medicine physician annotated the SPECT/CT images with lesion location. ROIs were drawn by an image analysis specialist, and the segmentation was reviewed by the nuclear medicine physician or a radiologist. The nuclear medicine physician or radiologist confirmed whether target lesion location matched the location information selected by the site. The same lesions selected to be quantified in Cycle 1 were followed in subsequent cycles (2 to 6 in phase I), if data were available.

### ROI quantitation and organ time-integrated activity coefficients (TIAC)

Calculation of time-integrated activity coefficient (TIAC, formerly called the mean residence time [MRT]), was performed for each delineated organ and up to five tumors for each patient in each study cycle in phase I. For each ROI (organ or lesion) and image acquisition (SPECT/CT), the concentration (Bq/mL), total activity (Bq), and ROI volume (mL) over time were computed with and without decay of ^177^Lu (a half-life of 9662.4 minutes was used). When decay correction was performed, end of infusion time was used. The concentration and total activity data over time were normalized by the known injected activity and converted from mL to L to arrive at units of fraction of injected activity (FIA) per liter (FIA/L) and FIA.

Organ quantitation of concentration and total activity differed depending on the ROI approach. When full organ segmentation was utilized (as is the case for lesions), the ROI concentration and total activity were used directly for the organ concentration and total activity. When fixed volume spheres or sub-organ ROIs were used, quantification of the total organ activity was performed by multiplying the ROI concentration by the appropriate phantom organ size. Kidney volumes were measured for each patient on the CT images and used for the organ’s mass of the ICRP-89 adult male or female phantom assuming a density of 1 g/mL. For all other organs, phantom organ weights from the ICRP-89 adult male or female phantom were linearly scaled by the ratio of the patient effective mass to the ICRP-89 adult male or female phantom mass (as appropriate). The patient effective mass was defined as the minimum of the actual patient mass and the patient’s maximum effective mass (M_max_) as described by Wahl et. al (3). M_max_ will be computed for each patient based on their height and sex as follows (3):

for males: **M_max_=1.4497×height (cm)**−**154.58**

for females: **M_max_=1.2444×height (cm)−126.79**

The actual patient mass was used to scale organ masses, unless it exceeded the M_max_ calculated for the patient, in which case, M_max_ was used for organ mass scaling.

Organ and lesion data were analyzed using multi-exponential time–activity curve (TAC) fitting. Biological clearance of the tracer was derived from a data-appropriate fit of each organ TAC or via established biokinetic models for organs that did not exhibit exponential clearance. Depending on the shape and number of time points of the TAC, the data were fitted with the sum of one or two exponentials or using a rise and fall exponential function. The appropriate function was selected based on Akaike Information Criterion analysis and visual inspection by an imaging scientist or senior analyst. Fitting was performed with in-house Python Programming Language (RRID:SCR_008394). TIACs were calculated through the sum of trapezoidal integration for non-fitted parts of the TAC, and analytical integration of the fitting curve from the start of the fit to infinity. Trapezoidal integration was applied in following cases:

- For all organs and lesions for which the data were fitted with a mono or bi exponential, trapezoidal integration was applied until the first imaging time point, assuming organ/lesion uptake at t=0 to be zero and linear increase until the first imaging time point.
- In cases where the curve fit did not encompass early time points or was not appropriately described at early time points, the TIAC was the sum of trapezoidal integration of the non-fitted part of the curve, and analytical integration from the start of the fit to infinity.
- If no appropriate fit was found (e.g. the fitting function had a slower decay than 177Lu physical decay), the TIAC was estimated using the sum of the trapezoidal integration of imaging measurements and physical decay from the last time point onward.

Integrals were computed after reincorporation of ^177^Lu physical decay. Functions and parameters used to fit the TAC were defined as follows:

- Mono-exponential with a in fIA; b in hours
  **MonoExp(t,a,b)=a×exp(−(1/b)×t)**
- Bi-exponential with a in fIA; b in hours; c in fIA; d in hours
  **BiExp(t,a,b,c,d)=a×exp(−(1/b)×t)+c×exp(−(1/d)×t)**
- Rise and Fall with a in fIA; b in hours; c in hours
  **riseFall(t,a,b,c)=a×exp(−(1/b)×t)−a×exp(−(1/(b+c))×t)**

The whole-body TIAC was derived from the whole-body TAC and assumed 100% whole-body uptake at the time of injection. TIAC of the remainder of the body was calculated by subtracting the cumulative organ TIACs from the whole body TIAC.

For the urinary bladder and the gastrointestinal tract, biokinetic models as implemented in Organ Level Internal Dose Assessment (OLINDA) software were used to account for urinary and alimentary tract excretion dynamics (4, 5). For all dosimetry patients, the TIAC of the urinary bladder contents was computed using the dynamic voiding bladder using the following parameters:

- The FIA cleared through the bladder was computed using 1−peak activity in the GI tract across time points. **Fraction=1**−**max(GI tract)**
- The urine clearance half-life was calculated by fitting the total body TAC with a mono-exponential function (or a bi-exponential function or alternative fitting approach, if appropriate).
- It was assumed that patients would void every 4 hours. If activity was seen in the gastrointestinal tract, the human alimentary tract (HAT) model was used. Peak total activity in the gastrointestinal tract across time points was used as the FIA cleared via the gastrointestinal tract.

### Dosimetry

The image- and gamma counter-derived TIACs were collated and input into OLINDA v2.2.3 or higher for the computation of organ and whole-body absorbed doses in addition to effective dose. For all target organs (**Table S4**), absorbed doses were computed at the individual level based on reference mass values for each target organ included in the ICRP-89 phantoms (6), and descriptive summary statistics were provided at the study site and global level.

Red marrow dosimetry was estimated following formalisms outlined by MIRD and European Association of Nuclear Medicine (EANM) (7, 8). A standard value for red marrow-to-blood activity concentration ratio (RMBLR) of 1.00 was used to estimate MRT (now known as TIAC) of the bone marrow from whole blood concentration, with total red marrow mass taken from the OLINDA ICRP-89 male or female model (as appropriate) and scaled to patient mass.

Lesion dosimetry was performed by segmenting the physical lesion volume on the diagnostic imaging CT or magnetic resonance imaging (MRI) closest to dosimetry and using this volume to contour the lesions on the SPECT and where applicable. The activity in the tumor VOI was corrected for partial volume effect by using a recovery coefficient using the tumor VOI volume derived from the CT/MRI and the RC curve determined at site set up using a NEMA phantom. The lesion activity as measured on the SPECT image in the segmented volume was multiplied by the recovery coefficient correction factor to correct the lesion activity for partial volume effect. The sphere model implemented in OLINDA was used to compute lesion dose, using the lesion volume as determined on the CT image. Tumor mass was derived using the lesion volume and a density of 1 g/mL.

## Tumor assessment imaging

### **CT and magnetic resonance imaging (MRI) acquisition parameters**

For patients tolerant of iodinated intravenous CT, contrast-enhanced CT (CECT) of the chest, abdomen, and pelvis were acquired (using the parameters outlined in **Table S5**). CECT was the standard imaging modality for Response Evaluation Criteria in Solid Tumors (RECIST) assessments, however, alternative imaging approaches were permitted where clinically necessary (e.g., CT without contrast or MRI with contrast), and RECIST measurements were applied to these modalities where feasible. For patients who were intolerant of, or at high risk of reaction to, iodinated intravenous CT contrast, unenhanced (no-contrast) CT of the chest and contrast-enhanced MRI of the abdomen and pelvis (using the parameters outlined in **Table S6**) were acquired. For patients who were intolerant of both iodinated and gadolinium-based contrast media, unenhanced CT of the chest and unenhanced MRI of the abdomen and pelvis were acquired. All brain imaging was acquired using MRI, unless MRI was contraindicated, in which case a CECT of the brain was acceptable. If MRI contrast was acceptable, imaging was performed according to the following schedule: pre-contrast: T1-weighted image (T1W) and T2-weighted image (T2W); post-contrast: T1W. If MRI contrast was contraindicated, an unenhanced MRI of the abdomen and pelvis was performed (according to the methods outlined in **Table S6**) and unenhanced T1W and T2W sequences were acquired.

### Tumor assessment criteria

Lesions were considered measurable according to the thresholds stipulated by the RECIST v1.1 publication (9):

- Measurable tumoral lesion
  - One or more linear dimensions can be accurately measured, and
  - ≥10 mm longest diameter in the axial plane.
- Measurable abnormal lymph node
  - ≥15 mm in the short axis (longest linear dimension perpendicular to the longest diameter).
- Special considerations for measurability
  - Linear measurement of irregularly shaped lesions should not cross normal tissue; however, the linear measurement may include areas of necrosis.
  - Although simple cysts should not be considered malignant nor selected, a cystic lesion that is thought to represent metastasis should be considered measurable if it meets the criteria for measurability.
  - Blastic bone lesions (increased bone density) should be considered non-measurable.
  - Lytic bone lesions (decreased bone density) or mixed lytic-blastic may be measurable if they contain a soft-tissue component that is visible on CT and they meet the criteria for measurability.

After identifying measurable lesions on the time-matched CT or MRI, corresponding lesions on the baseline ^68^Ga-FAP-2286 PET/CT were identified. Lesions were considered positive if they had activity greater than the adjacent background tissue. The number and location of each lesion was recorded.

After completing the baseline assessment, the same assessment was completed on the end of treatment CT or MRI and ^68^Ga-FAP-2286 PET/CT obtained after the last administration of ^177^Lu-FAP-2286. On the follow-up time point, the presence of lesions annotated at baseline was assessed and their current state (present or disappeared) was determined. Any new lesions were annotated.

# Supplementary references

1. Dewaraja YK, Frey EC, Sgouros G, Brill AB, Roberson P, Zanzonico PB, *et al.* MIRD pamphlet No. 23: quantitative SPECT for patient-specific 3-dimensional dosimetry in internal radionuclide therapy. *J Nucl Med* **2012**;53:1310–25.
2. Ljungberg M, Celler A, Konijnenberg MW, Eckerman KF, Dewaraja YK, Sjögreen-Gleisner K, *et al.* MIRD Pamphlet No. 26: Joint EANM/MIRD Guidelines for quantitative 177Lu SPECT applied for dosimetry of radiopharmaceutical therapy. *J Nucl Med* **2016**;57:151–62.
3. Wahl RL, Kroll S, Zasadny KR. Patient-specific whole-body dosimetry: principles and a simplified method for clinical implementation. *J Nucl Med* **1998**;39(8 suppl):14S–20S.
4. Thomas SR, Stabin MG, Chen CT, Samaratunga RC. MIRD Pamphlet No. 14 revised: A dynamic urinary bladder model for radiation dose calculations. Task Group of the MIRD Committee, Society of Nuclear Medicine. *J Nucl Med* **1999**;40:102S–23S.
5. International Commission on Radiological Protection. Human alimentary tract model for radiological protection. ICRP Publication 100. A report of The International Commission on Radiological Protection. *Ann ICRP* **2006**;36:25–327.
6. Basic anatomical and physiological data for use in radiological protection: reference values. A report of age- and gender-related differences in the anatomical and physiological characteristics of reference individuals. ICRP Publication 89. *Ann ICRP* **2002**;32:5–265.
7. Hindorf C, Glatting G, Chiesa C, Lindén O, Flux G. EANM Dosimetry Committee guidelines for bone marrow and whole-body dosimetry. *Eur J Nucl Med Mol Imaging* **2010**;37:1238–50.
8. Siegel JA, Thomas SR, Stubbs JB, Stabin MG, Hays MT, Koral KF, *et al*. MIRD pamphlet no. 16: Techniques for quantitative radiopharmaceutical biodistribution data acquisition and analysis for use in human radiation dose estimates. *J Nucl Med* **1999**;40:37s–61s.
9. Eisenhauer EA, Therasse P, Bogaerts J, Schwartz LH, Sargent D, Ford R, *et al*. New response evaluation criteria in solid tumours: revised RECIST guideline (version 1.1). *Eur J Cancer* **2009**;45:228–47.
