# Supplementary material for: Targeting Fibroblast Activation Protein with [177Lu]Lu-FAP-2286 in Patients with Advanced Solid Tumors in the Phase I LuMIERE Trial
Source: Clin Cancer Res. 2026 Jun 1;32(17):3757–67. doi: 10.1158/1078-0432.CCR-25-4356 (PMC13335097; doi:10.1158/1078-0432.CCR-25-4356)
Supplement: Supplementary file 4 [file ccr-25-4356_supplementary_table_s1_suppts1.docx]

**Table S1.** Prespecified toxicity/severity criteria for dosage-limiting toxicities.

| Toxicity | Any of the following criteria^a^ |
| --- | --- |
| Hematology | CTCAE Grade 4 neutropenia |
|  | ≥ CTCAE Grade 3 febrile neutropenia |
|  | CTCAE Grade 3 thrombocytopenia with bleeding |
|  | CTCAE Grade 4 thrombocytopenia |
|  | CTCAE Grade 3 anemia requiring a transfusion |
|  | CTCAE Grade 4 anemia |
| Renal | CTCAE Grade 3 creatinine increased with duration ≥72 hours |
|  | CTCAE Grade 4 creatinine increased |
|  | ≥ CTCAE Grade 3 proteinuria |
|  | ≥ CTCAE Grade 3 acute kidney injury |
| Hepatic | CTCAE Grade 3 total bilirubin with duration ≥72 hours |
|  | CTCAE Grade 4 bilirubin increased |
|  | CTCAE Grade 3 ALT and/or AST increased AND increase in total bilirubin ≥ Grade 1 with duration ≥72 hours |
|  | CTCAE Grade 4 ALT or AST increased |
| Other adverse events | ≥ CTCAE Grade 3 adverse events with the exception of:   - Fatigue with duration ≤72 hours - Diarrhea well controlled by systemic medication and with duration ≤72 hours - Nausea, vomiting, and/or dehydration well controlled by systemic medication and with a duration ≤72 hours |

Supportive care (e.g., antiemetics, steroid preparations, analgesics for pain control, normal saline for hydration) may be used at the investigator’s discretion and per institutional guidelines. 5-HT3 receptor antagonists should be avoided as a pre-medication for the first administration of ^177^Lu-FAP-2286 and should be used thereafter only when medically necessary. Alternative antiemetics, including NK1-receptor antagonists (e.g., aprepitant) may be used at the discretion of the investigator. Where erythropoietin, darbepoetin alfa, and/or hematopoietic colony-stimulating factors are used for the treatment of cytopenias, administration should be in accordance with standard of care and should follow institutional guidelines. Transfusion thresholds should follow institutional guidelines.

^a^ CTCAE v5.0 or higher will be used for grading all adverse events and laboratory abnormalities.

ALT, alanine aminotransferase; AST, aspartate aminotransferase, CTCAE, Common Terminology Criteria for Adverse Events.
