# Supplementary material for: Targeting Fibroblast Activation Protein with [177Lu]Lu-FAP-2286 in Patients with Advanced Solid Tumors in the Phase I LuMIERE Trial
Source: Clin Cancer Res. 2026 Jun 1;32(17):3757–67. doi: 10.1158/1078-0432.CCR-25-4356 (PMC13335097; doi:10.1158/1078-0432.CCR-25-4356)
Supplement: Supplementary file 5 [file ccr-25-4356_supplementary_table_s2_suppts2.docx]

**Table S2.** SPECT/CT parameters.

| Collimator type | Medium energy |
| --- | --- |
| Total number of projections | 120 |
| Projections per detector | 60 |
| Angular step | 3° |
| Total degrees of rotation per detector | 180° |
| Matrix | 128×128 |
| Time per projection | 20 seconds |
| Mode | Step and Shoot |
| Radius | Elliptical with Body Contour mode positioned as close as possible |
| Acquisition zoom | 1.0 |
| Photopeak energy window | 208.4 keV 20% (±10%) |
| Energy window range | ~187.6–229.2 keV |
| Scatter energy window (lower)^a^ | 177.1 keV 10% (±5%) |
| Scatter energy window range (lower)^a^ | ~166.7–187.6 keV |
| Scatter energy window (upper)^a^ | 239.6 keV 10% (±5%) |
| Scatter energy window (upper)^a^ | ~229.3–250.2 keV |
| CT^b^ | Low-dose; local standard procedures may be followed |

^a^ 10% lower and upper scatter window immediately adjacent to the main photopeak window. Actual keV values may differ slightly dependent on scanner make/model.

^b^ A low-dose CT scan was performed prior to beginning or immediately following the SPECT scan for attenuation correction. Institutional standard parameters were used for the low-dose CT.

CT, computed tomography; SPECT, single-photon emission computerized tomography.
