# Supplementary material for: Targeting Fibroblast Activation Protein with [177Lu]Lu-FAP-2286 in Patients with Advanced Solid Tumors in the Phase I LuMIERE Trial
Source: Clin Cancer Res. 2026 Jun 1;32(17):3757–67. doi: 10.1158/1078-0432.CCR-25-4356 (PMC13335097; doi:10.1158/1078-0432.CCR-25-4356)
Supplement: Supplementary file 6 [file ccr-25-4356_supplementary_table_s3_suppts3.docx]

**Table S3.** OLINDA source organs.

| Organs (only those organs with appreciable activity [>background via a qualitative assessment] are included in the analysis in addition to the ROIs indicated in bold) | | |
| --- | --- | --- |
| Adrenals | Rectum | Red marrow^b^ |
| Brain | Heart contents^a^ | Cortical bone |
| Breasts | Heart wall | Trabecular bone |
| Esophagus | Kidneys | Spleen |
| Eyes | Liver | Testes |
| Gallbladder contents | Lungs | Thymus |
| Left colon | Ovaries | Thyroid |
| Small intestine | Pancreas | Urinary bladder contents |
| Stomach contents | Prostate | Uterus |
| Right colon | Salivary glands | Total body/Remainder |

^a^ The time-integrated activity coefficient (TIAC) of the heart contents as a source region was estimated from the known concentration of activity in the blood and an estimate of patient-specific heart blood volume derived as the Organ Level Internal Dose Assessment (OLINDA) ICRP-89 phantom heart contents volume scaled by patient-to-phantom mass ratio. If appropriate blood gamma counter data were not available, the TIAC of the heart contents was estimated based on the single-photon emission computerized tomography (SPECT/CT) images.

^b^ The TIAC of the red marrow was estimated following formalisms outlined by Medical Internal Radiation Dose (MIRD) and European Association of Nuclear Medicine (1, 2). A standard value for red marrow-to-blood activity concentration ratio of 1 was used to estimate mean residence time of the bone marrow from whole blood concentration, with total red marrow mass taken from the OLINDA ICRP-89 male or female model (as appropriate) and scaled to patient mass. If appropriate blood gamma counter data were not available, the TIAC of the red marrow was estimated based on the SPECT/CT images.

ROI, region of interest.

**Supplementary references**

1. Hindorf C, Glatting G, Chiesa C, Lindén O, Flux G. EANM Dosimetry Committee guidelines for bone marrow and whole-body dosimetry. *Eur J Nucl Med Mol Imaging* **2010**;37:1238–50.
2. Siegel JA, Thomas SR, Stubbs JB, Stabin MG, Hays MT, Koral KF, *et al*. MIRD pamphlet no. 16: Techniques for quantitative radiopharmaceutical biodistribution data acquisition and analysis for use in human radiation dose estimates. *J Nucl Med* **1999**;40:37s–61s.
