# Supplementary material for: Targeting Fibroblast Activation Protein with [177Lu]Lu-FAP-2286 in Patients with Advanced Solid Tumors in the Phase I LuMIERE Trial
Source: Clin Cancer Res. 2026 Jun 1;32(17):3757–67. doi: 10.1158/1078-0432.CCR-25-4356 (PMC13335097; doi:10.1158/1078-0432.CCR-25-4356)
Supplement: Supplementary file 7 [file ccr-25-4356_supplementary_table_s4_suppts4.docx]

**Table S4.** OLINDA target organs.

| Target organs | | |
| --- | --- | --- |
| Adrenals | Rectum | Osteogenic cells |
| Brain | Heart wall | Spleen |
| Breasts^a^ | Kidneys | Testes^b^ |
| Esophagus | Liver | Thymus |
| Eyes | Lungs | Thyroid |
| Gallbladder wall | Ovaries^a^ | Urinary bladder wall |
| Left colon | Pancreas | Uterus^a^ |
| Small intestine | Prostate^b^ | Total body |
| Stomach wall | Salivary glands |  |
| Right colon | Red marrow |  |

^a^ Female phantom.

^b^ Male phantom.

OLINDA, Organ Level Internal Dose Assessment.
