# Supplementary material for: Targeting Fibroblast Activation Protein with [177Lu]Lu-FAP-2286 in Patients with Advanced Solid Tumors in the Phase I LuMIERE Trial
Source: Clin Cancer Res. 2026 Jun 1;32(17):3757–67. doi: 10.1158/1078-0432.CCR-25-4356 (PMC13335097; doi:10.1158/1078-0432.CCR-25-4356)
Supplement: Supplementary file 8 [file ccr-25-4356_supplementary_table_s5_suppts5.docx]

**Table S5.** CT acquisition parameters.

| Parameter | Brain | Chest, abdomen, and pelvis (CAP) | |
| --- | --- | --- | --- |
| Superior extent | Vertex | Superior extent of lung apices | |
| Inferior extent | Foramen magnum | Chest alone: including the entire posterior costophrenic recess | CAP: below the symphysis pubis |
| Slice thickness | Helical ≤5 mm (slice thickness of ≤3 mm preferred) | | |
| Spacing | Contiguous (0 mm spacing) | | |
| Algorithm | Standard (lung if required) | | |
| Oral contrast | Apply site standard of care | | |
| IV contrast | Follow institutional standard of care for use of intravenous CT contrast (tailor to the needs and limitations of each individual participant) | | |
| Breathing instructions | - CAP: single breath-hold if possible   or   - Chest and abdomen (through to the full inferior extent of the liver) in single breath-hold, followed by pelvis with quiet breathing | | |

CT, computed tomography.
