# Supplementary material for: Targeting Fibroblast Activation Protein with [177Lu]Lu-FAP-2286 in Patients with Advanced Solid Tumors in the Phase I LuMIERE Trial
Source: Clin Cancer Res. 2026 Jun 1;32(17):3757–67. doi: 10.1158/1078-0432.CCR-25-4356 (PMC13335097; doi:10.1158/1078-0432.CCR-25-4356)
Supplement: Supplementary file 9 [file ccr-25-4356_supplementary_table_s6_suppts6.docx]

**Table S6.** MRI acquisition parameters.

| Parameter | Brain | Abdomen and pelvis |
| --- | --- | --- |
| Superior extent | Vertex | Upper extent of the dome of the liver |
| Inferior extent | Foramen magnum | Below the symphysis pubis |
| Sequences | - T1W (axial) - T2W (axial) - FLAIR (axial) - T1W post-contrast (axial) or axial 3D T1W | - T1-weighted image (axial) (T1W) with FAT SAT - T2-weighted image (axial) (T2W) with FAT SAT - T1 post-contrast (axial) with FAT SAT |
| Slice thickness | 5 mm (≤3 mm preferred) | ≤5 mm recommended |
| Interslice gap (spacing) | 0 mm | 0 mm |
| Matrix | 256×192 (or higher) preferred | |
| IV contrast^a^ | Tailor to the needs or limitations of each individual patient. Do not use linear gadolinium agents. Follow site standard of care for use of MRI contrast in patients with renal insufficiency | |

T1W, T1-weighted image; T2W, T2-weighted image; FAT SAT, fat saturation; FLAIR (fluid attenuated inversion recovery)

^a^ For patients with acute or chronic severe renal insufficiency (glomerular filtration rate <30 mL/min), IV gadolinium contrast is contraindicated. Only non-contrast MRI scans are to be performed for such patients.

MRI, magnetic resonance imaging.
