# Supplementary material for: Targeting Fibroblast Activation Protein with [177Lu]Lu-FAP-2286 in Patients with Advanced Solid Tumors in the Phase I LuMIERE Trial
Source: Clin Cancer Res. 2026 Jun 1;32(17):3757–67. doi: 10.1158/1078-0432.CCR-25-4356 (PMC13335097; doi:10.1158/1078-0432.CCR-25-4356)
Supplement: Supplementary file 10 [file ccr-25-4356_supplementary_table_s7_suppts7.docx]

**Supplementary data – Table S7.** Representativeness of study participants.

| Cancer type(s)/subtype(s)/ stage(s)/condition | Advanced solid tumors |
| --- | --- |
| Considerations related to: |  |
| Sex | According to GLOBOCAN estimates, of the 20.0 million new global cancer cases in 2022, 51.5% (i.e. 10.3 million) were in males and 48.5% (i.e. 9.7 million) were in females (1). |
| Age | In the US, the median age at the time of cancer diagnosis (for cancers of any site) was 67 years between 2018 and 2022 (2). |
| Race/ethnicity | In the US, the age-adjusted incidence of cancer (for cancers of all sites) between 2018 and 2022 by race was highest among the American Indian/Alaskan Native population (497.8), followed by the White population (482.2), the Black population (467.7), and the Asian American/Pacific Islander population (311.2) (3). |
| Geography | Based on GLOBOCAN estimates, of the 20.0 million new global cancer cases in 2022, 49.2% were reported in Asia, 22.4% in Europe, 21.1% in the Americas, 5.9% in Africa, and 1.4% in Oceania (1). |
| Overall representativeness of this study | **Sex:** In phase I of LuMIERE, the sex distribution among the 27 patients with advanced solid tumors who were treated with ^177^Lu-FAP-2286 was 51.9% male and 48.1% female; this was representative of global incidence rates for cancer in males and females in 2022 (1).  **Age:** In phase I of LuMIERE, the median patient age was 60 years. This was lower than the median age at the time of cancer diagnosis in the US between 2018 and 2022 (67 years) (2). However, it was similar to the median age (59 years) of patients enrolled in phase I trials for metastatic cancer in the US between 2000 and 2018 (4).  **Race/ethnicity:** In phase I of LuMIERE, 63.0% of patients were White, 22.2% were Black/African American, and 11.1% were Asian; race was not reported for one patient (3.7%).  No American Indian/Alaskan Native patients were recruited, even though this population had the highest age-adjusted incidence rate in the US, relative to other racial groups (3); racial representativeness may have been limited by the demographics of patients surrounding the six medical centers in which phase I of LuMIERE was conducted.  Of note, a study of phase I trials for metastatic cancer conducted in the US between 2000 and 2018 (4) reported that the racial distribution of trial participants was 86.1% White, 6.2% Black, 4.5% Asian/Pacific Islander, and 0.3% American Indian/Alaskan Native. It found that phase I trials overrepresented White patients and underrepresented Black and Asian/Pacific Islander patients relative to incidence data from a US cancer registry (84.1% White, 11.8% Black, and 5.9% Asian/Pacific Islander). By comparison, LuMIERE phase I included a higher proportion of patients who were Black/African American and Asian than this study of phase I trials. |
|  | **Geography:** The LuMIERE phase I study was conducted in six medical centers in the US; therefore, it may not be representative of the overall global population with advanced solid tumors or with the distribution of solid tumors reported in any individual country/region.  **Summary:** Phase I of LuMIERE was designed to investigate the safety and tolerability of ^177^Lu-FAP-2286 monotherapy in heavily pretreated patients with a range of different solid tumor types. During the study, patients with pancreatic (n=9), colorectal (n=5), sarcoma (n=5), head and neck (n=3), and other (n=1 each) solid tumors were treated with ^177^Lu-FAP-2286. The study was not intended to be representative of the overall population with advanced solid tumors or any individual cancer type. |

# **Supplementary references**

1. Bray F, Laversanne M, Sung H, Ferlay J, Siegel RL, Soerjomataram I, *et al*. Global cancer statistics 2022: GLOBOCAN estimates of incidence and mortality worldwide for 36 cancers in 185 countries. *CA Cancer J Clin* **2024**;74:229–63.
2. National Cancer Institute, Surveillance, Epidemiology and End Results Program. Cancer Stat Facts: Cancer of Any Site [cited 2026 Mar 25]. Available from: <https://seer.cancer.gov/statfacts/html/all.html>.
3. Siegel RL, Kratzer TB, Wagle NS, Sung H, Jemal A. Cancer statistics, 2026. *CA Cancer J Clin* **2026**;76:e70043.
4. Dunlop H, Fitzpatrick E, Kurti K, Deeb S, Gillespie EF, Dover L, *et al*. Participation of patients from racial and ethnic minority groups in phase 1 early cancer drug development trials in the US, 2000-2018. *JAMA Netw Open* **2022**;5:e2239884.
