# Supplementary material for: Targeting Fibroblast Activation Protein with [177Lu]Lu-FAP-2286 in Patients with Advanced Solid Tumors in the Phase I LuMIERE Trial
Source: Clin Cancer Res. 2026 Jun 1;32(17):3757–67. doi: 10.1158/1078-0432.CCR-25-4356 (PMC13335097; doi:10.1158/1078-0432.CCR-25-4356)
Supplement: Supplementary file 11 [file ccr-25-4356_supplementary_table_s8_suppts8.docx]

**Supplementary data – Table S8.** Serious adverse events (^177^Lu-FAP-2286 safety set, N=27).

| Primary system organ class  Preferred term | All causality,  n (%) | Treatment related, n (%) |
| --- | --- | --- |
| Patients with ≥1 SAE | 7 (25.9) | 1 (3.7) |
| Gastrointestinal disorders |  |  |
| Abdominal pain | 2 (7.4) | 0 |
| Hepatobiliary disorders |  |  |
| Cholangitis | 1 (3.7) | 0 |
| Infections and infestations |  |  |
| Bacteremia | 1 (3.7) | 0 |
| Liver abscess | 1 (3.7) | 0 |
| Sepsis | 1 (3.7) | 0 |
| Spontaneous bacterial peritonitis | 1 (3.7) | 0 |
| Injury, poisoning, and procedural complications |  |  |
| Spinal compression fracture | 1 (3.7) | 0 |
| Metabolism and nutrition disorders |  |  |
| Dehydration | 1 (3.7) | 0 |
| Musculoskeletal and connective tissue disorders |  |  |
| Back pain | 1 (3.7) | 0 |
| Product issues |  |  |
| Device occlusion | 1 (3.7) | 0 |
| Respiratory, thoracic, and mediastinal disorders |  |  |
| Dyspnea | 1 (3.7) | 0 |
| Hemoptysis | 1 (3.7) | 1 (3.7) |
| Pulmonary embolism | 1 (3.7) | 0 |

Data represent patients for whom an SAE occurred within 42 days of ^177^Lu-FAP-2286 administration. Preferred terms are sorted by descending frequency for the “All causality” column.

SAE, serious adverse event.
