# Supplementary material for: Targeting Fibroblast Activation Protein with [177Lu]Lu-FAP-2286 in Patients with Advanced Solid Tumors in the Phase I LuMIERE Trial
Source: Clin Cancer Res. 2026 Jun 1;32(17):3757–67. doi: 10.1158/1078-0432.CCR-25-4356 (PMC13335097; doi:10.1158/1078-0432.CCR-25-4356)
Supplement: Supplementary file 12 [file ccr-25-4356_supplementary_table_s9_suppts9.docx]

**Supplementary data – Table S9.** Dosimetry of ^177^Lu-FAP-2286 in Cycle 1 (^177^Lu-FAP-2286 dosimetry set, N=24).

| Target | Observed absorbed dose following first administration (Gy/GBq) | | | | | Cumulative absorbed dose 55.5 GBq (Gy) (N=24) |
| --- | --- | --- | --- | --- | --- | --- |
|  | 3.70 GBq (n=3) | 5.55 GBq (n=6) | 7.40 GBq (n=4) | 9.25 GBq (n=11) | Overall (N=24) |  |
| Kidney | 0.22 (0.14) | 0.32 (0.22) | 0.43 (0.20) | 0.36 (0.15) | 0.35 (0.17) | 19 (9.7) |
| Red marrow | 0.034 (0.012) | 0.049 (0.013) | 0.043 (0.013) | 0.040 (0.009) | 0.042 (0.011) | 2.3 (0.63) |
| Adrenals | 0.037 (0.011) | 0.042 (0.018) | 0.033 (0.0028) | 0.036 (0.0086) | 0.037 (0.011) | 2.1 (0.63) |
| Brain | 0.034 (0.010) | 0.037 (0.018) | 0.027 (0.0021) | 0.031 (0.0092) | 0.032 (0.011) | 1.8 (0.63) |
| Breasts^a^ | 0.035 (0.013) | 0.026 | 0.027 (0.0021) | 0.033 (0.0076) | 0.032 (0.0077) | 1.8 (0.43) |
| Esophagus | 0.034 (0.010) | 0.038 (0.018) | 0.028 (0.002) | 0.031 (0.009) | 0.033 (0.011) | 1.8 (0.63) |
| Eyes | 0.034 (0.010) | 0.037 (0.018) | 0.027 (0.0021) | 0.031 (0.0092) | 0.032 (0.011) | 1.8 (0.63) |
| Gallbladder wall | 0.035 (0.011) | 0.044 (0.019) | 0.030 (0.0024) | 0.10 (0.23) | 0.067 (0.15) | 3.7 (8.5) |
| Heart wall | 0.046 (0.012) | 0.062 (0.019) | 0.049 (0.0028) | 0.047 (0.013) | 0.051 (0.014) | 2.8 (0.78) |
| Left colon | 0.077 (0.054) | 0.11 (0.12) | 0.038 (0.014) | 0.10 (0.051) | 0.091 (0.071) | 5.0 (3.9) |
| Liver | 0.013 (0.00083) | 0.027 (0.022) | 0.034 (0.030) | 0.032 (0.016) | 0.029 (0.019) | 1.6 (1.1) |
| Lungs | 0.030 (0.0019) | 0.042 (0.018) | 0.029 (0.012) | 0.049 (0.024) | 0.041 (0.020) | 2.3 (1.1) |
| Osteogenic cells | 0.033 (0.011) | 0.049 (0.020) | 0.037 (0.0095) | 0.036 (0.0076) | 0.039 (0.013) | 2.2 (0.72) |
| Ovaries^a^ | 0.039 (0.014) | 0.030 | 0.032 (0.0037) | 0.037 (0.0079) | 0.036 (0.008) | 2.0 (0.44) |
| Pancreas | 0.035 (0.011) | 0.039 (0.019) | 0.029 (0.0022) | 0.033 (0.0095) | 0.034 (0.012) | 1.9 (0.65) |
| Prostate^b^ | 0.032 | 0.043 (0.020) | 0.030 (0.0028) | 0.026 (0.0078) | 0.035 (0.015) | 1.9 (0.84) |
| Rectum | 0.077 (0.053) | 0.11 (0.11) | 0.040 (0.015) | 0.10 (0.049) | 0.090 (0.067) | 5.0 (3.7) |
| Right colon | 0.058 (0.034) | 0.077 (0.066) | 0.034 (0.0081) | 0.070 (0.028) | 0.064 (0.040) | 3.6 (2.2) |
| Salivary glands | 0.034 (0.010) | 0.038 (0.018) | 0.027 (0.002) | 0.031 (0.0091) | 0.032 (0.011) | 1.8 (0.64) |
| Small intestine | 0.039 (0.014) | 0.045 (0.023) | 0.030 (0.0032) | 0.038 (0.0096) | 0.039 (0.014) | 2.1 (0.79) |
| Spleen | 0.036 (0.011) | 0.039 (0.018) | 0.030 (0.0028) | 0.033 (0.0094) | 0.034 (0.011) | 1.9 (0.64) |
| Stomach wall | 0.035 (0.011) | 0.038 (0.018) | 0.028 (0.0021) | 0.032 (0.0094) | 0.033 (0.012) | 1.9 (0.64) |
| Testes^b^ | 0.030 | 0.040 (0.019) | 0.027 (0.0028) | 0.023 (0.0078) | 0.032 (0.015) | 1.8 (0.82) |
| Thymus | 0.034 (0.010) | 0.038 (0.018) | 0.028 (0.0021) | 0.032 (0.0094) | 0.033 (0.011) | 1.8 (0.63) |
| Thyroid | 0.034 (0.010) | 0.038 (0.018) | 0.027 (0.002) | 0.031 (0.0091) | 0.032 (0.011) | 1.8 (0.63) |
| Total body/remainder | 0.039 (0.012) | 0.043 (0.018) | 0.035 (0.0044) | 0.038 (0.0092) | 0.039 (0.011) | 2.2 (0.62) |
| Urinary bladder wall | 0.47 (0.074) | 0.42 (0.067) | 0.49 (0.12) | 0.53 (0.069) | 0.49 (0.088) | 27 (4.9) |
| Uterus^a^ | 0.041 (0.014) | 0.032 | 0.23 (0.28) | 0.058 (0.056) | 0.080 (0.11) | 4.4 (6.4) |

Data are mean (standard deviation).

^a^ 3.70 GBq (n=2), 5.55 GBq (n=1), 7.40 GBq (n=2), 9.25 GBq (n=8), overall (n=13), cumulative absorbed dose (n=13).

^b^ 3.70 GBq (n=1), 5.55 GBq (n=5), 7.40 GBq (n=2), 9.25 GBq (n=3), overall (n=11), cumulative absorbed dose (n=11).
